# MicroRNAs and long non-coding RNAs in the pathophysiological processes of diabetic cardiomyopathy: emerging biomarkers and potential therapeutics

**DOI:** 10.1186/s12933-021-01245-2

**Published:** 2021-02-27

**Authors:** Daniel Jakubik, Alex Fitas, Ceren Eyileten, Joanna Jarosz-Popek, Anna Nowak, Pamela Czajka, Zofia Wicik, Harald Sourij, Jolanta M. Siller-Matula, Salvatore De Rosa, Marek Postula

**Affiliations:** 1 Department of Experimental and Clinical Pharmacology, Centre for Preclinical Research and Technology CEPT, Medical University of Warsaw, Banacha 1B Str., 02-097 Warsaw, Poland; 2 Centro de Matemática, Computação e Cognição, Universidade Federal Do ABC, São Paulo, Brazil; 3 Division of Endocrinology and Diabetology, Department of Internal Medicine, Medical University of Graz, Graz, Austria; 4 Department of Cardiology, Medical University of Vienna, Vienna, Austria; 5 Division of Cardiology, Department of Medical and Surgical Sciences, “Magna Graecia” University, Catanzaro, Italy; 6 Cardiovascular Research Center, “Magna Graecia” University, Catanzaro, Italy; 7 Doctoral School, Medical University of Warsaw, 02-091 Warsaw, Poland

**Keywords:** miRNA, LncRNA, Diabetes mellitus type 2, Diabetic cardiomyopathy, Inflammation, Oxidative stress, Novel biomarker, Novel treatment, Novel therapy

## Abstract

The epidemic of diabetes mellitus (DM) necessitates the development of novel therapeutic and preventative strategies to attenuate complications of this debilitating disease. Diabetic cardiomyopathy (DCM) is a frequent disorder affecting individuals diagnosed with DM characterized by left ventricular hypertrophy, diastolic and systolic dysfunction and myocardial fibrosis in the absence of other heart diseases. Progression of DCM is associated with impaired cardiac insulin metabolic signaling, increased oxidative stress, impaired mitochondrial and cardiomyocyte calcium metabolism, and inflammation. Various non-coding RNAs, such as microRNAs (miRNAs) and long non-coding RNAs (lncRNAs), as well as their target genes are implicated in the complex pathophysiology of DCM. It has been demonstrated that miRNAs and lncRNAs play an important role in maintaining homeostasis through regulation of multiple genes, thus they attract substantial scientific interest as biomarkers for diagnosis, prognosis and as a potential therapeutic strategy in DM complications. This article will review the different miRNAs and lncRNA studied in the context of DM, including type 1 and type 2 diabetes and the contribution of pathophysiological mechanisms including inflammatory response, oxidative stress, apoptosis, hypertrophy and fibrosis to the development of DCM 
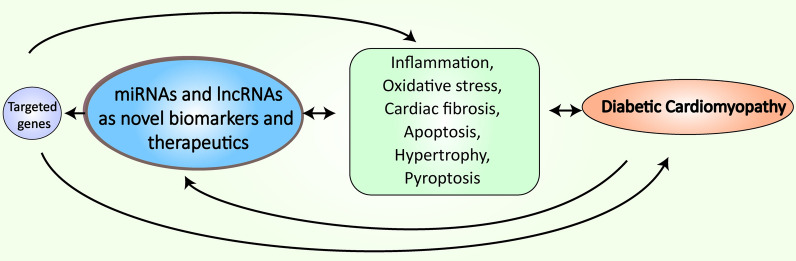
.

## Background

Diabetes mellitus (DM) is a disease characterized by chronic hyperglycemia and impaired metabolism of carbohydrates, proteins, and lipids caused by impaired insulin secretion and action. Approximately 463 million people aged 20–79 years are currently living with DM. The number of affected people is estimated to reach 578 million by 2030 according to the International Diabetes Federation. Cardiovascular diseases (CVDs) are the most common cause of mortality of diabetic patients [1]. There are two different forms of diabetes. Type 1 diabetes mellitus (T1DM) is a chronic disease due to lack of insulin hormone production from pancreatic β-cells. On the other hand, type 2 diabetes (T2DM) is characterized by high blood glucose and ineffective insulin response [2, 3].

Abnormal cardiac structure and function in patients with DM with no other CVDs i.e. coronary artery disease (CAD), valvular heart disease, and hypertension, is known as diabetic cardiomyopathy (DCM) [4]. DCM is associated with myocardial fibrosis and cardiac remodeling leading to impaired diastolic and systolic function, and consequently to heart failure (HF). The Framingham Heart Study showed the prevalence of HF increase of 2.4-fold in men and fivefold in women with DM in comparison to controls [5]. The development and progression of DCM are closely related to abnormal cardiac insulin metabolic signaling, increased oxidative stress, impaired mitochondrial, cardiomyocyte calcium handling and inflammation. Typically DCM has a long subclinical period [6, 7].

Since clear diagnostic criteria are still lacking, the diagnosis of DCM remains a challenge [8, 9]. Currently, there is a lack of specific histological property, biomarker, or clinical manifestation for the definitive diagnosis of DCM [8, 10]. The earliest changes observed in patients relate to myocardial fibrosis and left ventricular hypertrophy with subsequent systolic dysfunction, which develop in the early stages of diabetes [11, 12]. Although there are dedicated guidelines for the management of DM and HF as isolated conditions, there is insufficient guidance on caring for patients with both DM and HF [9, 13]. Currently, the most widely used test for DCM diagnosis is echocardiography. This approach allows the simultaneous detection of structural and functional changes in the myocardium and the exclusion of other potential causes of the disorder [11, 12, 14, 15]. On the other hand, the routine use of echocardiography for screening purposes appears to be uneconomical. Therefore, the situation requires the development of new blood-based diagnostic tools that will allow identification of patients with increased risk of DCM.

The current strategy for treating DCM is adequate control of DM, improvement of cardiovascular risk factors, including treatment of obesity and hypertension, and standard treatment of HF when required. Unfortunately, although DM has been known as an independent predictor of HF for many years, there is currently no individualized therapy to prevent HF and reduce the economic costs associated with treating these patients [16].

Inflammation is a complex response of an organism to different stimuli, which has two phases—acute and chronic. In the acute phase granulocytes, cytokines and acute-phase proteins (APPs) play a role in removing the inflammatory stimuli. Continuous inflammatory stimulation or impaired reaction to self-molecules leads to a chronic phase, in which an immune response can cause tissue damage and fibrosis. Chronic inflammation is considered to contribute to numerous diseases such as cerebrovascular diseases, atherosclerosis, cardiomyopathy, and DM [17]. There is increasing evidence that chronic inflammation is one of the main factors implicated in DM pathophysiology [18, 19].

Oxidative stress is an imbalance in the generation and elimination of reactive oxygen species (ROS) [20, 21]. ROS are highly unstable free radicals, produced mostly within mitochondria and endoplasmic reticulum. At normal levels, ROS take part in biological processes like immune response or cellular components maturation. On the other hand, high levels of free radicals can cause cell damage [22]. Oxidative stress plays a crucial role in the pathophysiology of numerous diseases including DM, in which glucose overload in mitochondria causes excessive ROS generation and mitochondrial dysfunction [23].

There are many well-known markers of inflammatory processes such as cytokines or APPs—CRP, amyloid A (AA) and amyloid P (AP), complement compounds, ceruloplasmin, or α2M [17]. However, novel and more precise biomarkers that will allow the early diagnosis of DCM are needed [15].

Pathophysiological mechanisms leading to DCM may also include impairment in microRNA (miRNA, miR) and long non-coding RNA (lncRNA) regulatory networks [6, 7, 24]. MiRNAs are a class of small, endogenous, non-coding RNA (ncRNA) molecules. They play a role in numerous biological processes like cell proliferation, differentiation, apoptosis, and metabolism, through the suppression or activation of gene expression. MiRNAs are stable and abundant in different body fluids, which makes them useful as potential novel biomarkers for DM and its complications [25–27]. LncRNAs are cell and tissue-specific transcripts consisting of more than 200 nucleotides (nt) that are not translated into proteins. LncRNAs are predicted to have many functions including transcript regulation, nuclear domains organization, and regulation of proteins or RNA molecules like miRNA. LncRNA through base-pairing interactions influence miRNAs abundance and activity [7, 28].

Recent reports indicate participation of ncRNAs in the pathogenesis of oxidative stress and inflammation related to several human disorders including DM complications [29, 30]. Also, it was shown that ncRNA may act as a promising tool to facilitate the development of therapeutic strategies and clinical management of patients with cardiomyopathy [31]. Obesity often accompanies DM. Independently of other cardiometabolic risk factors, it dysregulates inflammation-related ncRNAs. It was demonstrated that therapeutic manipulations of inflammation-related ncRNAs expression can potentially treat obesity-induced vascular complication [32, 33].

This review aims to provide a comprehensive overview of the current knowledge of diagnostic, prognostic, and treatment value of miRNA and lncRNA in the pathophysiological processes of DCM.

## The essential role of miRNAs in the regulation of DCM pathogenic processes

MiRNAs regulate protein synthesis, mostly through inhibition of mRNA. Synthesis of those molecules can be influenced by high glucose (HG) levels, thus miRNAs play a role in DM-related pathophysiological processes in the myocardium [34, 35]. Based on previous studies it was found that more than 300 different miRNAs are dysregulated in DCM. MiRNAs can modulate the oxidative stress response, influence the inflammatory processes and cardiomyocytes survival. Hence, they might be useful to treat and monitor DCM [36–38].

### The synthesis of miRNAs can be altered on different levels in the diabetic model

The previous study used the insulin2 mutant (Ins2+/−) Akita, a genetic mice model of T1DM to investigate the role of Dicer, a crucial enzyme for miRNA maturation and several miRNAs in DCM. The study showed that Dicer, numerous miRNAs and inflammatory cytokines (TNFα and cardiac IL-10) are associated with diabetes-induced HF. Dicer is an RNase III endonuclease plays role on pre-miRNAs maturation. The study found the upregulation of mRNA and protein levels of Dicer in diabetic mouse hearts compared to wild type [39]. Besides, many miRNAs, such as Let-7a, miR-345-3p, miR-433 and miR-455 were downregulated, while the only upregulated miRNA was miR-295. It is important to note that the study used only microarray analysis with no qRT-PCR validation. MiR-295 is typical for the embryonic stage, thus its upregulation in diabetic myocardium may indicate adaptive mechanisms to pathological conditions. Let-7a was also found downregulated in diabetic nephropathy by targeting PI3K/Akt signaling, an important pathway in the pathogenesis of insulin resistance and DCM development [40]. Moreover, miR-345 family was identified as an important regulator in children with the recent onset of T1DM. Importantly, in silico pathway analysis based on inferred miRNA target genes showed that PI3K/Akt, MAPK, and Wnt signaling pathways are related to T1DM [41]. Moreover, miR-433 was identified as a novel regulator of doxorubicin-induced cardiac fibrosis both in an animal model and in cardiac tissue from patients with dilated cardiomyopathy. Therefore further analyzes are needed to clarify its importance in DCM [42].

Another study pointed out the importance of miR-373 related to the MAPK signaling pathway. MiR-373 was significantly downregulated in diabetic mice cardiac tissue. In vitro analysis in rat cardiomyocytes exposed to HG and transfected by miR-373 showed that overexpression of miR-373 decreased MEF2C and hypertrophy. *MEF2C,* a transcription factor commonly found in heart tissue, seems to be a target gene for miR-373. Moreover, gene ontology analysis revealed the MAPK signaling pathway is the one most associated with dysregulated miRNAs in the diabetic mouse heart. Further in vitro inhibition of p38 MAPK reduced miR-373 expression, which suggests that miR-373 transcription may be regulated by p38 MAPK. Thus, p38 MAPK/miR-373/MEF2C was suggested to be a regulatory pathway in glucose-dependent cardiomyocyte hypertrophy [43]*.*

Upregulation of miR-19b, miR-27a, miR-34a, miR-125b, miR-146a, miR-155, miR-210, miR-221 and the downregulation of miR-1 in DCM by in vitro*/*in vivo analysis was demonstrated [44]. An interesting study performed by Costantino et al. [44] on a DM mouse myocardium, indicates the existence of hyperglycaemic memory, which means that even after normalization of glucose levels, the negative effects of hyperglycemia can persist. MiRNA profiling in heart samples revealed that despite intensive glycaemic control, miRNA signatures in diabetic myocardium were only partially reversible. Bioinformatic analysis showed that among dysregulated miRNAs, miR-221, miR-146a, miR-34a, miR-210, miR-19b, miR-125b, miR-27a, and miR-155 were associated with oxidative stress. Among them, miR-221 was upregulated in the diabetic myocardium suggesting a key role of miR-221 in the progression of diabetic myocardial damage after obtaining normoglycemia. The study results also suggest that miR-34a may be a mediator linking DM and cardiac aging [44]. Moreover, glycaemic control failed to restore the underexpressed antifibrotic miRNAs, including miR-1, which is known to play an important role in cardiac dysfunction under hyperglycemia [44, 45]. Additionally, it was previously shown that the expression of miR-210 is induced in diabetic ischaemic HF patients [46]. The existence of metabolic memory has been proposed previously, although its mechanisms were not clearly explained [47]. MiRNAs help to determine why the diabetic cardiovascular complications progression is not halted by the normalization of glucose levels. The results of the study done by Costantino et al. suggest oxidative stress-related miRNAs as potential novel therapeutic targets. Inhibition of those miRNAs (miR-221, miR-146a, miR-34a, miR-210, miR-19b, miR-125b, miR-27a, miR-155) may lead to the reduction of adverse effects of hyperglycaemic memory in the heart [44] (Table 1**)**.

**Table 1:** Evaluating miRNAs as a treatment approach in diabetic cardiomyopathy

Refs.	miRNA Down- or up-regulation	Regulated genes	Pathophysiological mechanism	Species, material, method	Conclusion
*Upregulated miRNAs in DCM*					
[44]	↑miR-19b ↑miR-27a ↑miR-34a ↑miR-125b ↑miR-146a ↑miR-155 ↑miR-210 ↑miR-221	*P27/* *mTOR* *calcineurin/NFAT*	Oxidative stress Hypertrophy Apoptosis	STZ-induced diabetes model* In vivo, (C57/B6 mice were used) *Diabetes was induced by a single high dose of STZ, 180 mg/kg, via intraperitoneal injection. Hyperglycemia was defined as 3 random blood glucose levels > 13.9 mmol/l	MiRNAs regulating redox signalling pathways (miR-221, miR-146a, miR-34a, miR-210, miR-19b, miR-125b, miR-27a, miR-155) were persistently dysregulated after normalization of blood glucose levels MiR-221 modulates cardiac hypertrophy via *P27/mTOR, calcineurin/NFAT* signaling pathway
[37]	miR-21	*PPARα,* *Nrf2*	Oxidative stress Inflammation Apoptosis	STZ-induced diabetes model* In vivo*, *in vitro, mice, rats (C57/B6J mice with generated LAZ3 knock down model by retro-orbital venous injection of AAV9-shLAZ3, NRCM cells transfected with adenovirus (Ad-) to overexpress LAZ3 were used), and were infected with Ad-LAZ3 and co-cultured with miR-21 mimic, miR-361 mimic or miR-155 mimic *Diabetic animal model was established by intraperitoneal STZ injection at a dose of 50 mg/kg for 5 consecutive days. One week after the final STZ injection, fasting blood glucose was measured. Diabetes was defined as FBG ≥ 16.6 mmol/L	LAZ3 is decreased in DCM mouse hearts and rats cardiomyocytes. LAZ3 inhibition enhanced miR-21, miR-155, miR-361 expression levels. LAZ3 modulates the *PPARα/Nrf2* pathway while downregulating miR-21. Knockdown of LAZ3 induces a severe inflammation, oxidative stress and apoptosis in rat cardiomyocytes. Contrarily, the increase of LAZ3 inhibits HG provoked myocardial injury
[38]	↑miR-30d	*FOXO3*	Inflammation Pyroptosis Apoptosis	STZ-induced diabetes model* In vivo*, *in vitro, (Wistar rats and NRCM cells were used and were transfected with miR-30d mimics (AMO-mir-30d) and NC miRNA (AMO-NC) using X-treme GENE siRNA Transfection Reagent) *Diabetic Wistar rats were injected intraperitoneally with 35 mg/kg/d of STZ for 3 days. Diabetes was defined as glucose level ≥ 16.7 mM	Enhanced miR-30d inhibited *FOXO3* resulting in decreased ARC and therefore increased CASP-1, and pro-inflammatory cytokines IL-1β, IL-18 levels confirming its role in DCM progression
[102]	↑miR-30d	*KLF9/VEGFA*	Autophagy	STZ-induced diabetes model* in vivo*,* rats Sprague Dawley rats were fed with a high-fat diet during 10 week and injected with STZ 30 mg/kg/d for 5 consecutive days. Diabetes was defined as at least two glucose levels ≥ 16.7 mmol/L or one fasting glucose ≥ 8.0 mmol/L	SGLT-2 inhibitors can regulate the autophagy level in diabetic rats through the miR-30d/KLF9/VEGFA pathway, thereby improving cardiac function
[60]	↑miR-150-5p	*Smad7*	Fibrosis Inflammation	HG- induced diabetes model* In vitro, rats cardiac fibroblasts *Neonatal cardiac fibroblasts were treated with HG (30 mM, 50 mM) DMEM in the presence of the TGF-β1 inhibitor or NF-kB inhibitor for 24 h and were transfected with miR-15-5p mimics and miR-15-5p inhibitor (AMO-150-5p) inhibitor using X-treme GENE siRNA Transfection Reagent	*Smad7* is the direct target of miR-150-5p Inhibition of miR-150-5p could prevent NF-kB induced inflammation and TGF-β1/Smad-triggered cardiac fibrosis by targeting *Smad7*
[50, 162]	↑miR-155	*SHIP-1* *SOCS1* *BCL6*	Inflammation Apoptosis Fibrosis	STZ-induced diabetes model* In vivo*, *in vitro (C57/BL6 mice, RAW 264.7 cells were transfected with miR-155 mimics and miR-155 inhibitors) *C57/BL6 mice were injected intraperitoneally with a low dose of STZ (50 mg/day/kg) for 5 consecutive days, hyperglycemia was defined as glucose level > 22 mmol/L after 2 weeks	Overexpression of miR-155 caused disbalance between proinflammatory (M1) and anti-inflammatory (M2) macrophages causing impaired cardiac hemostasis MiR-155 promoted M1 polarization and therefore enhanced inflammation AuNP-mediated anti-miR-155 delivery stimulated M2 polarization, therefore diminished cells apoptosis, inflammation, fibrosis and enhanced cardiac function in OVX mice
[104]	↑miR-195	*BCL2* *SIRT*	Oxidative stress Apoptosis Hypertrophy	T1DM STZ-induced diabetes model* T2DM model by using db/db mice In vitro*, *in vivo*,* (C57BL/6 mice, db/db mice cardiomyocytes and ECs were infected with adenoviral vectors containing miR-195 (Ad-miR-195) or β-gal as a control *Diabetes was induced in mice by consecutive peritoneal injections of STZ 50 mg/kg/d for 5 days. Diabetes was defined as glucose level ≥ 15 mmol/l at 72 h after STZ injection, to silence miR-195 expression in hearts, we used antimiR-195 miR construct (miRZip-195)	*M*iR-195 expression was increased and levels of its target proteins *BCL2* and *SIRT1* were decreased in STZ-induced type 1 and db/db type 2 diabetic mouse hearts. In vivo inhibition of miR-195 led to the improved coronary blood flow, increased both *BCL2* and *SIRT1* expression and resulted in the downregulation of TNFα, IL-1β and CASP-3 activity
[93]	↑miR-503	*Nrf2*	Oxidative stress Apoptosis	STZ-induced diabetes model* In vitro*, *in vivo*,* (Male Wistar rats, rats primary cardiomyocytes, HEK293T transfected with miR-503 mimics, NC mimics) *Rats in the DM group were fed with a high-fat diet per day. 30 mg/kg/d of STZ was injected intraperitoneally for 3 consecutive days. Diabetes was defined as glucose level ≥ 16.7 mM	Increased levels of miR-503 impact DCM progression. Decreased miR-503 is involved in decreasing DCM progression via CPDT which is activating *Nrf2*/ARE pathway and *Nrf2* is target gene for miR-503
*Downregulated miRNAs inDCM*					
[45]	↓miR-1	*Junctin*	Oxidative stress	T1DM STZ-induced diabetes model* In vivo*, *in vitro*,* (Wistar rats and mice C2C12 cells were used) *Diabetes was induced by single intraperitoneal injection of STZ (50 mg/kg)	MiR-1 directly targets junctin and suppresses its expression. Decreased levels of miR-1 in HG-conditions result in increased expression of junctin Overexpression of junctin induced cardiac hypertrophy and arrhythmia via adaptive changes in Ca2 + handling
[44]	↓miR-1	*Fibulin-1*	Oxidative stress Hypertrophy Fibrosis	STZ-induced diabetes model* In vivo, (C57/B6 mice were used) *Diabetes was induced by a single high dose of STZ, 180 mg/kg, via intraperitoneal injection. Hyperglycemia was defined as 3 random blood glucose levels > 13.9 mmol/l	MiR-1 replacement treatment is suggested to play a crucial role in cardiac hypertrophy and fibrosis by targeting *Fibulin-1*
[103]	↓miR-9	*ELAVL1 CASP-1*	Inflammation Pyroptosis	In vitro*, human* (Human diabetic heart tissue obtained from failing hearts at the time of transplantation, diabetic human cultured cardiomyocytes were co-transfected with control or miR-9 mimic or miR-9 inhibitor H9C2 Raw 264.7	Expression of miR-9 is downregulated in human diabetic failing heart. Upregulation of miR-9 reduces *ELAVL1, CASP-1* and IL-1β expression in human cardiomyocytes
[121]	↓miR-21	*Gelsolin*	Oxidative stress Hypertrophy	In vivo*, *in vitro, (C57BL/Ks mice transfected with rAAV-tnt-GFP, rAAV-tnt-miR-21, and rAAV-tnt-miR-21-TUD; H9c2, HEK293 cells, HL-1 cardiac muscle cell line from AT-1 mouse and human cardiomyocytes were used)	MiR-21 is downregulated in cardiomyocytes in diabetic mice model MiR-21 directly targeted *gelsolin* and inhibited the *gelsolin* pathway Mimic miR-21 treatment reduced myocardial hypertrophy in diastolic dysfunction db/db mice by diminishing ROS levels and increasing eNOS forced release in db/db mice
[94]	↓miR-22	*SIRT1*	Oxidative stress, Apoptosis	T1DM STZ-induced diabetes model* In vivo*, *in vitro*,* mice (C57BL/6 mice, embryonic cardiac myoblast cell line (H9c2 cells)—cultured in high glucose conditions were used) *Diabetes was induced in mice by consecutive peritoneal injections of STZ 50 mg/kg/d for 5 days. Diabetes was defined as glucose level ≥ 16.6 mmol/l	MiR-22 was found downregulated in the hearts of diabetic mice. Increased miR-22 expression enhanced cardiac function in DCM by increasing SOD levels and decreasing ROS, MDA levels Additionally, miR-22 is able to block Bax/Bcl-2, cl-CASP-3/CASP-3 and cl-CASP-9/CASP-9 in HG-treated H9c2 cells
[80]	↓miR-30c	*PGC-1β*	Oxidative stress Apoptosis	T1DM STZ-induced diabetes model* In vivo*, *in vitro*,* (db/db C57BL/Ks mice, H9c2 cells, HEK293T primary NRC) Db/db mice had single tail vein injection of the rAAV-miR-30c or rAAV-anti-miR-30c randomly, H9c2 cells were first transfected with PGC-1β siRNA or miR-30c, and then transfected with pTK-PPREx3-Luc plasmid. HEK293T cells were co-transfected with appropriate pMIR construct, pRL-TK plasmid with miR-30c mimics or negative controls	In the diabetic mouse model, miR-30c levels were downregulated. Upregulation of miR-30c decreased *PGC-1β* expression and therefore lipotoxicity and cardiac dysfunction suggesting its cardioprotective role *PGC-1β* knockdown inhibited PPRAα transcriptional activity in diabetic mouse hearts
[86]	↓MiR-30c	*Cdc42, Pak1*	Cardiomyocytehypertrophy	STZ-induced diabetes model* in vitro*, *in vivo*,* rats, human cardiac tissue *Diabetes in Wistar rats was induced by two injections of STZ 30 mg/kg/d a week apart, preceded by 4 weeks of high-fat-diet	Downregulation of miR-30c mediates prohypertrophic effects of hyperglycemia in DCM by upregulation of Cdc42 and Pak1 genes
[87]	↓MiR-30c	*p53, p21*	Hypertrophy, apoptosis	STZ-induced diabetes model* in vitro*, *in vivo*,* rats, human cardiac tissue *Wistar rats were fed high-fat-diet for 4 weeks, followed by two injections of STZ 30 mg/kg/d, a week apart	Dysregulation of miR-30c and miR-181a may be involved in upregulation of the p53–p21 pathway in DCM
[88]	↓MiR-30c	*BECN1*	Autophagy	in vitro*, *in vivo, human plasma, mice, rat cardiomyocytes Plasma obtained from healthy controls (n = 28), DM patients (n = 26), patients with chronic HF (n = 16), and patients with both DM and chronic HF (n = 15) Diabetic cardiomyopathy db/db mice and non-diabetic control C57BL/Ks mice	Downregulation of miR-30c and subsequent activation of BECN1 promotes autophagy, contributing to the pathogenesis of DCM
[51]	↓miR-126	*ADAM9*	Inflammation Apoptosis	In vitro*,* human, mice (Human heart tissue obtained from failing human hearts at the time of transplantation, mouse bone marrow derived macrophages (BMM) from non-diabetic and diabetic (db/db) mice, RAW 264.7 cells cultured in hyperglycemia (HG; 35 mM) conditions, BMM diabetic (db/db) cells were used)	Inhibition of miR-126 attenuate efferocytosis via upregulating *ADAM9* Increased *ADAM9* causes proteolytic cleavage of MerTK and inactive form of sMER Downregulation of MerTK phosphorylation diminished efferocytosis of apoptotic cells and reduced inflammation
[112]	↓MiR-133a	*SGK1, IGF1R*	Hypertrophy	STZ-induced diabetes model* in vitro*, *in vivo*,* mice, neonatal rat myocytes * C57BL/6 J mice injected STZ 50 mg/kg/d 3 consecutive days. Diabetes was defined as blood glucose level > 20 mmol/L on two consecutive days	Mir-133a mediates novel glucose-induced mechanism regulating gene expression and cardiomyocyte hypertrophy in diabetes
[113]	↓MiR-133a	*ERK1/2, SMAD-2*	Fibrosis	STZ-induced diabetes model* in vivo, mice Four types of animals: wild-type non-transgenic, non-transgenic with 2 months of (STZ)-induced diabetes, non-diabetic cardiac miR-133a transgenic and cardiac miR-133a transgenic with STZ-induced diabetes * mice were injected by STZ 65 mg/kg *i.p* for three consecutive days	In diabetic mice with cardiac-specific miR-133aa overexpression, cardiac fibrosis was significantly decreased. Furthermore, cardiac miR-133a overexpression prevented ERK1/2 and SMAD-2 phosphorylation. MiR-133a could be a potential therapeutic target for diabetes-induced cardiac fibrosis and related cardiac dysfunction
[66]	↓miR-142-3p	*Smad*	Fibrosis	in vitro*,* Primary human aortic endothelial cells (HAECs) were used	miR-142-3p could attenuate high glucose induced endothelial-to-mesenchymal transition in HAECs, the mechanism of which may at least partly involve blocking TGF-β1/Smad signaling pathway. This might provide a potential therapeutic target for DCM in the future
[34]	↓miR-144	*Nrf2*	Oxidative stress, Apoptosis	T1DM STZ-induced diabetes model* In vivo*, *in vitro*,* (C57BL/6 mice and mice cardiomyocytes incubated with 33 mmol/l glucose (HG) for 48 h were used and were transfected with miR-144 mimic or with miR-144 inhibitor) *C57/BL6 mice were injected intraperitoneally with STZ solution (150 mg/kg). Diabetes was defined as glucose level ≥ 16.7 mM	MiR-144 level was lower in heart tissues of STZ-induced diabetic mice and in cardiomyocytes cultured in HG conditions. However, further inhibition of miR-144 demonstrated both diminished oxidative stress and apoptosis resulting in enhanced myocardial function via increased *Nrf2* which is the main modulator of cellular reaction to oxidative stress
[71]	↓miR-146a	*IRAK, TRAF6* *NFKB* *IL6* *TNFA* *IL1B*	Inflammation	STZ-induced diabetes model* In vitro, human, HCMECs** in vivo*,* mice, primary mouse cardiac endothelial cells (MHECs) *C57BL/6 X CBA/J transgenic mice with miR-146a overexpression, Diabetic animals were induced by 5 intraperitoneal injections of STZ (50 mg/kg) on consecutive days **Human cardiac microvascular endothelial cells (HCMECs) were incubated with 25 mM glucose (HG) for 48 h. HCMECs were transfected with miRIDIAN miR-146a mimic or antagomir (20 nmol/L) using the transfection reagent Lipofectamine2000	Decreased miR-146a expression resulted in increased IL-6, TNFα, IL-1β, MCP-1, NF-κB, Col1α1, Col4α1 through *TRAF/IRAK*/ NF-κB pathway and the overexpression of miR-146a reversed this effect suggesting its cardioprotective role
[59]	↓miR-203	*PIK3CA* *PI3KT/Akt*	Oxidative stress Hypertrophy Fibrosis Apoptosis	STZ-induced diabetes model* In vivo* (*C57BL/6 mice) *C57/BL6 mice were injected intraperitoneally with STZ solution (50 mg/kg) for 5 consecutive days	*PIK3CA* is involved in progression of DCM *PIK3CA* is directly targeted via miR-203 and impacts *PI3K/Akt* signaling pathway Increased miR-203 expression and inhibition of *PI3K/Akt* pathway decreased cardiac fibrosis in diabetic mice model
[43]	↓miR-373	*MEF2C*	Hypertrophy	STZ-induced diabetes model* in vitro*, *in vivo, mice, neonatal rat myocytes * diabetes in C57/BL6 male mice was induced by a single injection of STZ, 150 mg/kg. Diabetes was defined as blood glucose level ≥ *18.6 mmol/L* on two consecutive days	Overexpression of miR-373 decreased the cell size, and reduced the level of its target gene MEF2C. miR-373 expression was regulated by p38, the member of MAPK subgroup which were specifically upregulated in cardiomyocyte hypertrophy during hyperglycemia

↑ / ↓indicates the up/down regulation of the miRNAs determined in the HG conditions / diabetic heart model

*Ad* adenovirus, *ADAM9* ADAM metallopeptidase domain 9, *AP-1* activator protein 1, *ARC* activity regulated cytoskeleton associated protein, *ARE* antioxidant response element, *AT-1* angiotensin II receptor type 1, *AuNP* gold nanoparticle, *Bax* Bcl-2-associated X protein, *Bcl2* B-cell lymphoma 2, *BCL6* BCL6 transcription repressor, *BMM* bone marrow-derived macrophages, *Ca* calcium, *CASP-1* caspase 1, *c-Fos* Fos proto-oncogene, *CFs* cardiac fibroblasts, *Col1α1* collagen Type I alpha 1, *Col4α1* collagen type IV alpha 1, *CPDT* 5,6-dihydrocyclopenta-1,2-dithiole-3-thione, *db/db* diabetic, *DCM* diabetic cardiomyopathy, *ECs* endothelial cells, *ELAVL1* ELAV like protein 1, *eNOS* endothelial NOS, *FBG* fasting blood glucose, *FOXO3* Forkhead box O3, *GFP* green fluorescent protein, *hCMECs* human cerebral microvascular endothelial cells, *HEK293T* human embryonic kidney cells, *HF* heart failure, *HG* high-glucose, *ICM* ischemic cardiomyopathy, *IL* interleukin, *IRAK* interleukin-1 receptor-associated kinase, *kg* kilogram, *L* litre, *LAZ3* lymphoma-associated zinc finger 3, *LV* left ventricle, *MCP-1* monocyte chemoattractant protein-1, *MDA* malondialdehyde, *MerTK* MER proto-oncogene, tyrosine kinase, *MHECs* mouse heart endothelial cells, *mg* milligram, *miRNA, miR* microRNA, *mM* millimolar, *mmol* millimole, *MMP-9* matrix metallopeptidase 9, *mTOR* mammalian target of rapamycin, *NC* negative control, *NFAT* nuclear factor of activated T-cells, *NF-κB* nuclear factor kappa-light-chain-enhancer of activated B cells, *NRCM* neonatal rat cardiomyocytes, *Nrf2* nuclear factor erythroid 2-related factor 2, *OVX* ovariectomized, *pcDNA* p-complementary DNA, *PGC-1β* peroxisome proliferator-activated receptor gamma coactivator 1-alpha, *PI3KT* phosphoinositide 3-kinase, *PI3K/Akt* phosphoinositide 3-kinase and protein kinase B, *PIK3CA* phosphoinositide 3-kinase catalytic subunit alpha, *PPARα* peroxisome proliferator-activated receptor-alpha, *pRL-TK* thymidine kinase promoter-Renilla luciferase reporter plasmid, *ROS* reactive oxygen species, *SHIP-1* phosphatidylinositol-3,4,5-trisphosphate 5-phosphatase 1, *siRNA* small interfering RNA, *SOCS1* the suppressor of cytokine signaling–1, *SOD* superoxide dismutase, *Sirt* Sirtuin, *Smad7* SMAD family member 7, *sMER* soluble MER, *STZ* streptozocin, *T1DM* Type 1 diabetes mellitus, *TGF-β1* transforming growth factor β1, *TNFα* tumor necrosis factor-alpha, *TRAF* TNF receptor associated factor

In conclusion, HG conditions can influence the synthesis of all miRNAs via the modulation of dicer. The function of this enzyme depends on the myocardial impairment stage. Additionally, changes in gene expression can be reversed only partially. These findings make miRNA-genes interactions even more complex and indicate the great importance of identifying novel therapeutic approaches that could potentially prevent the development of DCM.

### MiRNAs can modulate macrophages function and phenotypes

Macrophages play an important role in the regulation of inflammatory processes. Classically, activated M1 cells can produce pro-inflammatory cytokines, while alternatively activated M2 cells are important for the resolution of inflammation [48]. In inflammatory disorders miR-155 is known to be upregulated and the administration of antagomiR-155 (miR-155 inhibitor) was observed to decrease the cardiac infiltration by inflammatory mediators, reduce myocardial damage, and improve cardiac function [49]. Estrogen deficiency increased inflammation in DCM mice due to the over-infiltration by M1 macrophages (pro-inflammatory type) [50]. However, the aggravation of DCM caused by estrogen deficiency was prevented by treatment with gold nanoparticle-conjugated antagomiR-155, which induced M2 macrophages infiltration and ameliorated the structure and function of the heart. It is suggested that miR-155 inhibition therapy could serve as a promising approach to improve cardiac function in DCM [50].

**Fig. 1:**
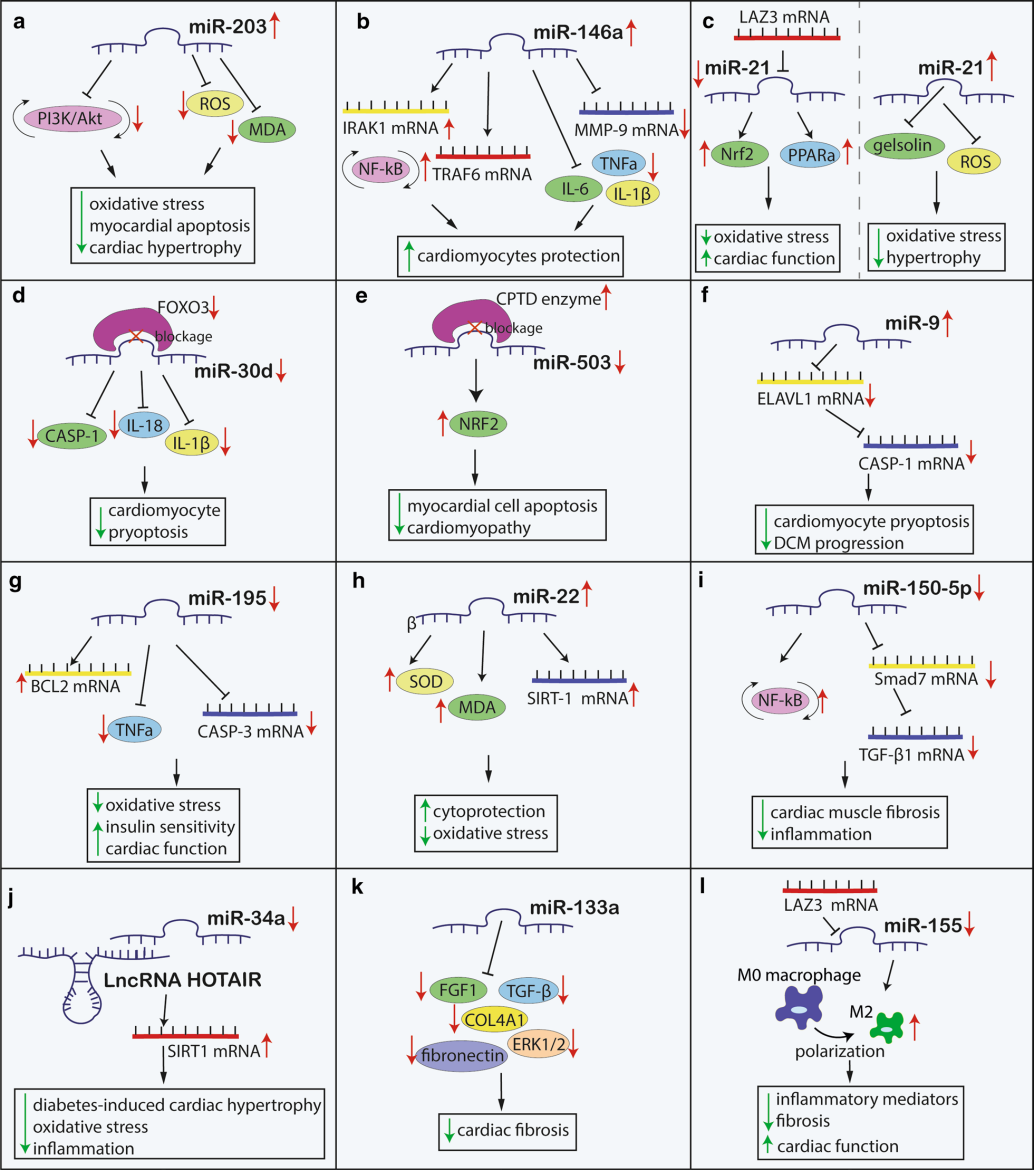
The possible therapeutic mechanism of miRNAs-contributed to the oxidative stress, inflammation and cardiomyocytes function process in diabetic cardiomyopathy. *Bcl-2* B-cell lymphoma 2, *CASP* Caspase, *Col4α1* collagen type IV alpha 1, *CPDT* 5,6 Dihydrocyclopenta-1,2-dithiole-3-thione, *DCM* diabetic cardiomyopathy, *ELAVL1* ELAV like protein 1, *FGF1* fibroblast growth factor 1, ERK1/2 extracellular signal-regulated kinases 1 and 2, *FOXO3a* Forkhead box O3, *HOTAIR* HOX transcript antisense intergenic RNA, *IL* interleukin, *IRAK1 *interleukin-1 receptor-associated kinase 1, *LAZ3* lymphoma-associated zinc finger 3, *LncRNA* long non-coding RNA, *MDA* malondialdehyde, *MiR* MicroRNA, *MMP-9* matrix metalloproteinase 9, *NF-κB* nuclear factor kappa-light-chain-enhancer of activated B cells, Nrf2: nuclear factor erythroid 2-related factor 2, *PI3K/Akt* phosphoinositide 3-kinase and protein kinase B, *PPARα* peroxisome proliferator-activated receptor-alpha, ROS reactive oxygen species, *SIRT-1* Sirtuin 1, *SMAD7* SMAD family member 7, *SOD* superoxide dismutase, *TGF-β1* transforming growth factor β1, *TNFα* tumor necrosis factor-alpha, *TRAF6* TNF receptor associated factor 6

Efferocytosis, phagocytic clearance of the apoptotic cells, is impaired in macrophages of DCM patients. MiR-126 was found to modify macrophage-mediated phagocytosis of apoptotic myocytes [51]. Under hyperglycemic conditions, the expression of miR-126 in macrophages was downregulated, which was accompanied by increased expression of its target gene, *ADAM9.* ADAMs are membrane-anchored enzymes that are involved in a variety of biological processes including cytokine and growth factor shedding, cell migration, as well as inflammatory response [52]. Similarly, overexpression of miR-126 diminishes efferocytosis impairment. Thus, enhancing this pathway by pharmacological treatment that will induce the expression of miR-126, has a potential to improve cardiac muscle function after injury and under inflammatory conditions accompanying DM [51] (Fig. 1) (Table 1).

### The importance of NF‐κB induced inflammation and TNFα mediated processes in the development of DCM

NF‐κB is a family of transcription factors that regulate multiple biological processes, including, inflammatory response, cell survival and cell cycle progression [53, 54]. PI3K/Akt pathway can activate NF‐κB induced inflammation [55]. PI3K/Akt signaling also represents a pivotal pathway in the pathogenesis of insulin resistance and DCM development. It regulates multiple biological processes, including apoptosis, cell growth, and proliferation of cardiomyocytes [56]. PI3KT/Akt activates platelets in response to multiple stimuli and subsequently may aggravate fibrosis of the heart muscle as activated platelets can have profibrotic action by releasing TGF-β1 and inducing platelet-fibroblast conjugation. TGF-β1 plays a key role in cardiac fibrosis and platelets can contain high concentrations of TGF-β1. Importantly, studies showed that platelet-derived TGF-β1 promoted ventricular fibrosis in a mouse model and atrial fibrosis in cell culture [57, 58].

Yang et al. [59] aimed to evaluate the role of miR-203 and PI3KT/Akt pathway in the progression of DCM in a rodent model. It was found that miR-203, which is downregulated in diabetic mice, directly targets *PIK3CA* and can downstream PI3KT/Akt pathway. In cardiomyocytes, miR-203-mediated inhibition of PI3K/Akt was found to be related to reduced cardiac hypertrophy, fibrosis, and myocardial apoptosis. Interestingly, the upregulation of miR-203 reduced the levels of oxidative stress biomarkers, such as MDA and ROS, in cardiomyocytes. The study suggests that the upregulation of miR-203 might be a promising treatment strategy for inhibiting the progression of DCM via PI3KT/Akt cascade [59].

In HG conditions cardiac fibroblasts exhibit markedly increased IL‐1β production and NF‐κB activity. This is accompanied by a significantly upregulated expression of miR-150-5p and downregulation of its target gene expression—*Smad7 *[60]. *Smad7* is a shear-stress induced gene, the expression of which can be enhanced via NF‐κB signaling pathway. *Smad7* suppresses TGF‐β1 signaling, which results in the suppression of its anti-inflammatory actions [61]. The inhibition of miR-150-5p attenuates cardiac muscle fibrosis and inflammation mediated by NF‐κB signaling TGF‐β1/Smad pathways. HG-treated cardiac fibroblasts manifest also significantly elevated fibrotic markers and extracellular matrix (ECM) proteins: CTGF, FN, α‐SMA, Col‐I, Col‐III [60]. Importantly, it was previously shown that miR‐150‐5p plays a crucial role in nonclassical monocyte generation, development of B and T lymphocytes, inflammatory cytokine production, and vascular remodeling and fibrosis [62–65]. In lymphocytes, a direct target of miR-150-5p—c-myb is responsible for the regulation of hematopoietic stem cells. It was also found that c-myb is involved in miR-150-mediated ROS-induced cardiomyocytes apoptosis and injury [65]. It was shown that cardiac remodeling may be reversed by miR-150-5p knockdown and aggravated by its overexpression, thus inhibition of miR-150-5p may become a promising target for DCM treatment [60]**.**

Moreover, miR-142-3p was found to be a direct regulator of TGF-β1, a mediator essential for endothelial-to-mesenchymal transition (EMT) process, which plays an important role in myocardial fibrosis. The expression of miR-142-3p in human aortic endothelial cells (HAECs) exposed to HG levels was declining in a dose- and time-dependent manner. Further evaluation showed that miR-142-3p inhibits EMT induced by HG levels through blocking of TGF-β1/Smad pathway. MiR-142-3p/TGFβ1/Smad axis was suggested to become a possible target in DCM therapy [66]*.* Similarly, miR-700 was found to modulate cardiac fibrosis by interacting with TGF-β3 and Col1α1 in the heart [67]. Previously, Shen et al. demonstrated the upregulation of miR-700 in diabetes-induced cardiac hypertrophy by using microarray analysis in an STZ-induced T1DM animal model. Importantly, the study used a bioinformatic tool and found 28 putative target genes for miR-700. However, further analysis with qRT-PCR validations is needed to confirm the upregulation of this promising miRNA in DCM [43, 68].

MiR-146a has a regulatory relationship with components of the NF-κB signaling pathway, which acts as a key mediator in hyperglycemia and inflammation [69, 70]. Several studies have indicated the important role of miR-146a in the pathogenesis of myocardial injury and DCM [44, 71–73]. Importantly, miR-146a was suggested as a predictive biomarker of HF [73]. Myocardial injury increases the expression of several pro-inflammatory mediators (IL-6, MCP-1, TNFα), contributing to the progression of cardiac muscle remodeling, which leads to its irreversible dysfunction and in consequence to HF [74, 75]. Impaired miR-146a expression was found associated with subclinical inflammation and insulin resistance in T2DM patients [76]. Downregulated miR-146a levels were also found in the hearts of diabetic mice [71]. Interestingly, endothelial cells (ECs) were the main cell type that exhibited decreased miR-146a levels, while cardiomyocytes remained unaltered [71]. Along with diminished miR-146a expression, the level of cardiac functional abnormalities, including defective cardiac contractility as well as inflammatory markers and ECM proteins (IL-6, TNFα, IL-1β, MCP-1, NF-κB, Col1α1, Col4α1) were increased in the hearts of DM wild type mice. However, these changes were not observed in the diabetic transgenic mice with overexpression of miR-146a. In vitro studies with isolated human cardiac ECs revealed glucose-induced upregulation of *IRAK1* and *TRAF6*, which are specific NF-κB regulators and targets of miR-146a [71]. An increased level of miR-146a transcripts is accompanied by a decreased level of c-Fos mRNA and diminished activity of AP-1, a c-Fos-containing transcription factor complex. Downregulation of the c-Fos/AP-1 signaling by miR-146a inhibits MMP-9 activity, which is involved in cardiac remodeling. Thus, the overexpression of miR-146a appears to play a protective role against cardiomyocytes injury and may be a novel therapeutic approach for the prevention of CVD, however further studies are needed [71, 72]**.**

MiR-223, which is downregulated in DM, is associated with inflammatory response [39, 77]. MiR-223 targets *Mef2c* and, what follows, inhibits the proliferation of myeloid progenitor cells, suppresses granulocytes differentiation and activation. Thus, the miR-223 downregulation leads to an excessive inflammatory response [78]. The suppression of miR-223 could result from attenuated IL-10 transcription. Moreover, due to insufficient inhibitory function of IL-10, elevated levels of TNFα can be observed [39]. Upregulation of TNFα may lead to enhancing TNFα-induced tissue factor pro-coagulation activity in ECs [77]. Importantly, in left ventricular biopsies of patients with T2DM miR-223 was found to mediate cardiac function by regulating the Glut4 expression and cardiomyocyte glucose metabolism [79]. Moreover, miR-223 was found significantly downregulated in left ventricular cardiac biopsies in diabetic ischaemic HF patients [46] (Fig. 1) (Table 1).

### MiRNAs can modulate cell response to oxidative stress acting through PPARα and Nrf2 transcription factors

There is a growing evidence that oxidative stress plays an important role in the progression of myocardial dysfunction in DM [34, 80]. Therefore, it is essential to search for mechanisms underpinning the association between oxidative stress and cardiac function. Nrf2 is an increasingly interesting transcriptional factor acting as a key regulator of oxidative stress genes. In DM models, the activation of Nrf2 is enhanced due to excessive production of oxidizing agents [81]. Fatty acids activated transcriptional factors such as PPARs can demonstrate anti-inflammatory activity and are confirmed to downregulate the expression of proinflammatory genes through transrepressive mechanisms [82]. Both Nrf2 and PPARs play a key role in establishing cellular antioxidative defense systems. Moreover, several studies strongly suggest the existence of reciprocal regulation of Nrf2 and PPARs signaling pathways, which mutually reinforces their expression [83, 84]. Activation of PPARα pathway may result in secondary changes in the oxidative stress state, which may lead to Nrf2 activation via PGC-1α [85].

Recent data indicate that miR-30c may be involved in transcriptional activity of PPARα [80]. Interestingly, in T1DM diabetic model, miR-30c levels were downregulated leading to the higher expression of its direct target—PGC-1β, which by acting on PPARα causes metabolic disturbances, lipotoxicity in the heart, and excessive ROS production [80]. Importantly, miR-30c expression was found reduced in cardiac tissue and plasma collected from DCM patients [86–88] Another study showed that forced overexpression of miR-30c in HG-induced cardiomyocytes was correlated with downregulation of prohypertrophic genes—*Cdc42* and *Pak1* and attenuation of cardiomyocyte hypertrophy, while anti-miR-30c treatment had the opposite effect [86]. Moreover, miR-30c may act synergistically with miR-181a in the modulation of the p53-p21 pathway important for cardiomyocyte apoptosis and hypertrophy in DCM. HG-induced cardiomyocytes transfection by miR-30c and miR-181a caused a decrease of p53 and p21 expression, ANP protein levels, and significantly attenuated hypertrophy. The effect was more potent when both miRNAs were overexpressed than for miR-30c or miR-181 alone [87]. Additionally, transfection of recombinant adeno-associated virus 9 (rAAV9)-mediated miR-30c in DCM mice resulted in cardiomyocyte miR-30c overexpression in DCM model resulted in the increased left ventricular ejection fraction, reduced left ventricle mass, and fractional shortening in comparison controls [88]. MiR-30c seems to be a multidirectional player in DCM. Therefore, the overexpression of miR-30c is hypothesized to attenuate cardiac dysfunction and appears to be a promising therapeutic target in DCM [80].

Another pathway regulating PPARα and Nrf2 activation is related to *LAZ3*, which is a protein-encoding gene that acts as a transcriptional repressor and regulates inflammation by interfering with NF-κB signaling [37, 89]. *LAZ3* was found decreased in diabetic mouse hearts and cardiomyocytes of rats [37]. Upregulation of *LAZ3* inhibits the miR-21 expression, which targets PPARα. Silencing of *LAZ3* leads to the increased expression of miR-21 and subsequently to the decreased PPARα and Nrf2 activation, resulting in an impaired response to the oxidative stress. Thus, the downregulation of the PPARα-Nrf2 signaling pathway by the overexpression of miR-21 results in the impaired cardiomyocytes function. Therefore, treatment targeting miR-21 inhibition may be beneficial for DCM management [37]. In line with these observations, a cardiac release of miR-21 has been recently reported in an unselected cohort of patients with non-ischemic cardiomyopathy, including DCM [90].

Downregulated miR-144 was found in diabetic cardiomyocytes in the regulation of oxidative stress. MiR-144 can directly target Nrf2 [34]. Interestingly, although miR-144 levels were reduced in T1DM diabetic mice model and in HG conditions in cultured cardiomyocytes, the administration of miR-144 mimic reduced the expression of Nrf2 proteins and augmented ROS formation, whereas miR-144 inhibitor enhanced Nrf2 expression and decreased ROS generation. This effect was not observed in normal glucose conditions. Thus, blocking miR-144 as a therapeutic intervention may decrease oxidative stress in HG condition [34]. On the other hand, Tao et al. [91] have found conflicting results. Plasma miR-144 decreased markedly in diabetic patients with cardiac dysfunction Although miR-144 was found also decreased in HG-induced cardiomyocytes and in an STZ-induced diabetic model, the overexpression of miR-144 was shown to protect the heart from the hyperglycemia-induced injury. To clarify the role of miR-144 in DCM, it may be necessary to generate mice with cardiomyocyte-specific miR-144 knockout or overexpression, at different stages of hyperglycemic cardiac injury [91].

The upregulation of miR-503 may be related to the increased diabetic cardiac dysfunction and Nrf2 activation can be enhanced through the phase II enzyme inducer –CPDT, an enzyme complex, which initiates the expression of antioxidative enzymes and plays a crucial role in the protection against oxidative stress [92, 93]. It has been hypothesized that CPDT may lead to a similar effect in diabetic heart dysfunction and that the downregulation of miR-503 can decrease the DCM development. For these purposes, Miao et al. [93] used CPDT as an intervention agent to investigate its correlation with miR-503 in DCM. CPTD treated diabetic rats presented with decreased expression of miR-503 and increased levels of its target—Nrf2 as well as other detoxification enzymes such as MDA and HO-1, when compared to a non-treated diabetic group. The data suggest that CPTD has a protective effect because of its ability to inhibit miR-503 expression and, as a result, to increase *Nrf2* expression, which can result in the diminished myocardial cell apoptosis and reduced development of cardiomyopathy [93].

Cardioprotective role is postulated regarding downregulation of miR-22 which is also directly linked with oxidative stress [94]. Excessive oxidative stress leads to the production of ROS, such as superoxide or hydrogen peroxide, which can cause DNA mutation, consequently resulting in cell injuries. On the other hand, superoxide dismutase (SOD) is an enzyme and can be effective as a potent antioxidant [95, 96]. MiR-22, the levels of which are decreased in diabetic myocardium in T1DM STZ-induced diabetes model, was found to target *SIRT1* leading to the upregulation of SIRT1 protein expression [94]. It was shown that the overexpression of miR-22 can decrease the levels of ROS, MDA, and increase SOD, indicating its cytoprotective properties. Administration of miR-22 had a positive effect on blood glucose levels, ejection fraction, left ventricular end-diastolic pressure, and cardiac tissue apoptosis in a diabetic animal model. Moreover, miR-22 was unable to inhibit the oxidative stress injury when *SIRT1* was knocked down, suggesting its protective effect being mediated by SIRT1 expression. Overall, this study shows that miR-22 administration can decrease oxidative stress injury by upregulation of SIRT1, thus miR-22 can be a potential therapeutic target for diabetic patients with cardiac insufficiency [94].

Altogether, in DM the modulation of levels of different miRNAs in the myocardium can be observed. Downregulated expressions of miR-30c, miR-22 and miR-144 and upregulated expressions of miR-503 were observed in in vivo and in vitro models of DCM. Those miRNAs influence the antioxidative action of Nrf2 transcription factor and impair its ability to prevent the adverse effects of oxidative stress due to ROS, which are synthesized in excessive amounts in DM (Fig. 1) (Table 1).

### MiRNAs can modulate the activation of cardiomyocyte pyroptosis and apoptosis via different pathways

Pyroptosis is an inflammatory form of programmed cell death triggered by CASP-1, which causes cardiac dysfunction due to decreased cell survival and increased pro-inflammatory cytokines, such as IL-1ß and IL-18 [97, 98]. Activation of these both interleukins is controlled by miR-30d, which is upregulated in HG conditions directly targeting *FOXO3a *[38]. MiR-30d mediates *FOXO3a* downregulation and results in decreased *ARC* and, what follows—increased *CASP-1* activation and inflammatory cytokines (IL-1ß and IL-18) secretion. It subsequently leads to the formation of the inflammasome complex, which induces the cardiomyocyte pyroptosis. Conversely, the miR-30d knock-down by its antisense inhibitor may suppress the process. Therefore, it is suggested that treatment targeted at blocking miR-30d expression may prove to be advantageous in DCM management [38]. Moreover, ARC was reported to protect cardiomyocytes in oxidative stress through inhibition of CASP-2-mediated apoptosis [99]. ARC also seems to attenuate the ischemia/reperfusion (I/R) injury and drug-induced cardiotoxicity [100, 101]. Besides that, in a rodent model, miR-30d showed also an anti-autophagic effect by regulating the KLF9/VEGFA pathway. Its knockdown prevented the aggravation of cardiac dysfunction in diabetic rats. Furthermore, SGLT2 treatment was associated with decreased miR-30d expression and improved cardiac function in DCM rats [102].

Another protein that plays an important role in pyroptosis-induced inflammatory processes is ELAVL1, which stabilizes TNFα mRNA. An increased concentration of CASP-1 proinflammatory enzyme, IL-1β proinflammatory cytokine along with an overexpression of ELAVL1 was found in human diabetic cardiomyocytes [103]. It was shown that ELAVL1 deficiency counteracts TNFα induced canonical pyroptosis via NLRP3, IL-1β and CASP-1 suppression. Moreover, ELAVL1 is a direct target of miR-9, the expression of which is significantly downregulated under hyperglycaemic conditions in human diabetic hearts. Inhibition of miR-9 can lead to the upregulation of ELAVL1 and CASP-1. On the other hand, overexpression of miR-9 reduces ELAVL1, CASP-1 and IL-1β expression in human cardiomyocytes and prevents cardiomyocyte pyroptosis. Thus, miR-9 may act as a potential target to reduce the DCM progression [103].

Apoptosis is another crucial factor triggering HF. It is a programmed self-eliminating process of dead cells occurring also in injured cardiomyocytes under different conditions e.g. ischemia. In T1DM and T2DM models, the upregulation of miR-195 has been confirmed to impact signaling pathways involved in oxidative stress-induced apoptosis. Furthermore, miR-195 was found to target *BCL2* and *SIRT1,* the expression of which was observed to be decreased in diabetic rat cardiomyocytes. Moreover, miR-195 levels are elevated in diabetic hearts in animal models, thus the inhibition of its targets and more intense apoptosis of cardiomyocytes can be observed. The knockout of miR-195 increased the levels of *SIRT1* and *BCL-2* and significantly improved cardiac function and coronary circulation but did not reduce myocardial fibrosis. Furthermore, inhibition of miR-195 reduced ROS production, protein oxidation and CASP-3 activity, indicating the role of miR-195 in oxidative stress-related cell injury. Moreover, the silencing of miR-195 inhibited TNFα mRNA and protein expression and improved insulin sensitivity [104]. Altogether, in HG conditions the downregulation of miR-9 and the upregulation of miR-30d and miR-195 can be observed. Modulations of their levels lead to enhanced pyroptosis and apoptosis in diabetic hearts and the progression of DCM (Fig. 1) (Table 1).

### The role of miR-1, miR-133a and miR-21 in DCM

Over the past decade, miR-1 and miR-21 have been some of the most frequently studied miRNAs in the CVD area, especially in the CAD, acute coronary syndrome and HF field [105, 106]. Evaluations revealed that these molecules are confirmed to impact signaling pathways involved in atherosclerosis, hypertrophy, myocardial remodeling and fibrosis [107]. Additionally, both miR-1 and miR-21 are hypothesized to be crucial targets for a new CVD treatment approach [108].

Junctin is a component of the ryanodine receptor Ca2 + release channel complex. It has been proved that the overexpression of junctin induces cardiac hypertrophy and arrhythmia via adaptive changes in Ca2 + handling [109, 110]. MiR-1, which is significantly downregulated in diabetic cardiomyocytes in the T1DM model of DCM, directly targets junctin and suppresses its expression [45]. Decreased levels of miR-1 in HG-conditions result in increased expression of junctin. Thus, the study suggests that miR-1 plays an important role in cardiac dysfunction under hyperglycemia. Indeed, miR-1 has been shown to contribute not only to the regulation of DCM, but also it was found to play a role in arrhythmias, myocardial infarction, myocardial hypertrophy, cardiomyocyte differentiation, and cell reprogramming [111]. It may be hypothesized that myocardial-specific miRNAs significantly contribute to DM-induced cardiomyocytes injury, and the intervention with antioxidant treatment controls the level of miRNAs including miR-1 and its target protein junctin, which can have a cardioprotective effect against DM-induced injury [45].

Abundantly expressed in normal cardiac tissue miR-133a was significantly downregulated in DCM mice. An in vitro study showed that miR-133a inhibited *SGK1* and *IGF1R* upregulation induced by HG levels leading to the depletion of ANP, BNP, and transcription factors MEF2A, and MEF2B as well as the attenuation of cardiomyocyte hypertrophy [112]. Furthermore, overexpressed miR-133a decreased mRNAs of fibrosis biomarkers such as fibronectin, FGF1, TGF-β, and COL4A1 and may prevent myocardial extracellular matrix (ECM) accumulation, presumably by preventing ERK1/2 and Smad2 phosphorylation. Thus, the lower expression of miR-133 associated with hyperglycemia may attenuate cardiac fibrosis [113]. In addition, miR-133a participates in cardiac contractility by targeting and downregulating tyrosine aminotransferase, an enzyme catabolizing tyrosine, a substrate for norepinephrine synthesis. In DCM rats, miR-133a deficiency was associated with decreased heart contractility. MiR-133a treatment resulted in increased expression of the β-adrenergic receptor, normalization of norepinephrine levels, and as a result, contractility improvement [114]. In transgenic diabetic mice with cardiac miR-133a overexpression (Akita/miR-133aTg) myocardial fibrosis, hypertrophy and impaired contractility were reduced when compared to standard Akita mice. Akita/miR-133aTg also had a normal lipid accumulation in heart tissue, whereas standard Akita exhibited lipid deposits [115]. Thus, miR-133a may prevent metabolic heart remodeling related to DM-induced lipotoxicity. In the plasma of DM patients and a mimic model of insulin resistance, increased miR-133a levels were associated with higher myocardial steatosis. It was shown that the lipid-loaded cardiomyocytes release exosomes rich in miR-133a. Therefore, miR-133a was proposed as a diagnostic biomarker of subclinical DCM [116].

MiR-21 is recognized as one of the most studied miRNAs that control myocardial remodeling [117]. However, it should be noted that the role of miR-21 in cardiac disease appears controversial. It was shown that miR-21 is upregulated in failing myocardium in humans and in an animal model, while interfering miR-21 expression with inhibitors prevents cardiac fibrosis in a mouse model of pressure overload [117, 118]. Moreover, upon pressure overload, cardiac dysfunction was only prevented in mice with miR-21 deficiency in nonmyocyte cardiac cells, but not in mice with global or myocyte targeted deletion of miR-21 [117]. Additionally, as mentioned above in neonatal rat cardiomyocytes incubated in HG conditions, it was shown that LAZ3 protects against cardiac remodeling by decreasing miR-21 [37]. Additionally, another in vitro study showed that HG conditions promoted the proliferation and collagen synthesis of rat cardiac fibroblasts, which was mediated by increased expression of miR-21 [119]**,**

It appears that the effect of miR-21 differs depending on cell type and disease condition. In cardiac myocytes the overexpression of miR-21 protects against ROS-induced damage via its target gene *PDCD4*, whereas miR-21 deficiency in fibroblasts protects against cardiac dysfunction and myocardial fibrosis [117, 120]. Also, the effect of miR-21 on ROS production and its role on cardiac myocytes in DCM associated diastolic dysfunction was studied [121]. Increased levels of cardiac oxidative stress biomarkers, which were found in diabetic mice model cardiomyocytes, were reversed by miR-21 treatment and overexpression of phospho-Akt and phospho-eNOS. Therefore, miR-21 inhibits cardiac hypertrophy via decreasing ROS levels and increasing bioavailable nitric oxide via gelsolin and thus may have potential therapeutic role [121]. Study conducted by Dai et al. suggested that the overexpression of miR-21 may be a promising therapeutic target for treatment of DCM [121]. On the other hand, due to the conflicted results, it can be concluded that miR-21 may have a different role depending on the tissue or cell type and disease condition, thus further human studies are needed to clear out this discrepancy (Table 1).

## LncRNAs

LncRNAs are non-protein-coding RNAs, that are at least 200 nt in length [7]. LncRNAs participate in many biological processes, such as modulation of various pathways linked with the oxidative stress and inflammatory processes, which influence DCM development, severity of myocardial I/R injury, cardiac hypertrophy, HF or diabetic vascular complications [7, 122]. Moreover, lncRNAs can act as miRNA sponges, meaning that they prevent the regulatory functions of miRNAs by binding to them and hindering interactions with their target [122].

### LncRNAs mediate cardiomyocytes apoptosis induced by high glucose

Cardiomyocyte apoptosis occurs in response to different pathological stimuli in DCM. As a consequence, it leads to the remodeling and fibrosis of the heart muscle, thus impairing its contractile function [123]. Several studies indicated the role of lncRNAs in pathomechanisms associated with deteriorating heart function in diabetic models [124, 125].

LncRNA H19, which plays a role in maintaining cardiac function and development of DCM, was known previously for its involvement in carcinogenesis [126]. It was shown that the expression of H19 was markedly downregulated in the myocardium of diabetic rats [127]. Moreover, transfection with H19 siRNA decreases the expression of miR-675. LncRNA H19 and H19-derived miR-675 downregulates its target gene *VDAC1*, which influences cardiomyocyte apoptosis and plays a crucial role in the progression of cardiac muscle dysfunction. Additionally, LV systolic and diastolic functions were found to be impaired in diabetic models along with exacerbated inflammation and oxidative stress. However, the overexpression of H19 reverses this effect [127].

In another study [128], authors sequenced 400 lncRNAs associated with ROS generation, which may potentially lead to the deterioration of cardiac function and apoptosis. In HG-treated primary rat cardiomyocytes ROS production was upregulated along with an increased level of apoptotic cardiomyocytes. However, among sequenced lncRNA, in further functional studies it was only the silencing of lncRNA NON-RATT007560.2 that decreased the ROS formation and apoptosis [128]. Importantly, it was found that NON-RATT007560.2 may have binding sites for miR-208a [128] which is known for its association with maladaptive cardiac remodelling in diabetic myocardium of T2DM patients [129].

Those studies suggest that the modulation of lncRNA expression may ameliorate cardiac dysfunction and apoptosis-related progression of cardiomyopathy, and may be a promising therapeutic strategy for the treatment of DCM.

### LncRNAs regulate cardiac remodeling via TGF-β-mediated NLRP3 pathway

Kcnq1ot1, which is a lncRNA, has been linked with pathophysiological mechanisms of multiple disorders associated with cardiac dysfunction [130, 131]. The Kcnq1ot1 expression was found to be elevated in the blood serum of diabetic patients, cardiac tissue of a T1DM STZ-induced diabetes mice model, and HG-induced cardiomyocytes along with activation of pyroptosis and fibrosis in DCM models [132, 133]. The increased expression of Kcnq1ot1 is followed by collagen deposition and induced TGF-β1, p-Smad2 and p-Smad3 expression was found. As a consequence, the activation of fibrotic formation and cardiac remodeling lead to the deterioration of LV function. Moreover, immunohistochemical analysis of mouse cardiac tissue showed that high Kcnq1ot1 levels contribute to NLRP3 inflammasome activation as well as significantly elevated expression of proinflammatory mediator-IL-1β and pro-apoptotic mediators-CASP-1 and GSDMD-N, thereby demonstrating the role of Kcnq1ot1 in mediating pyroptosis under HG conditions. On the other hand, the silencing of Kcnq1ot1 expression significantly improved cardiac function, reduced remodeling and pyroptosis via miR-214-3p and CASP-1 axis, and TGF-β1/Smads pathway [132].

In another study authors obtained replicated results [133]. In line with the above-mentioned findings, Kcnq1ot1 was enhanced in AC16 human myocardial cells in HG conditions, and followed by an increase of NLRP3, CASP-1, IL-1β, and IL-18 expression. It was shown that Kcnq1ot1 impacted post-acute myocardial infarction (AMI) I/R myocardial injury by regulating *Adipor1*. Additionally, Kcnq1ot1 knockdown promoted QT interval prolongation via *Kcnq1* gene inhibition. Transfection with small interfering RNA (siRNA) downregulated the level of those pyroptosis markers, reversed DNA fracture, ameliorated vimentin expression, and reduced Ca^2+^ overload. Silencing Kcnq1ot1 promoted the decrease of *CASP-1* mRNA and protein expression, as well as the increase of miR-214-3p level. What is more, the inhibition of CASP-1 in primary cardiomyocytes resulted in reduced Ca^2+^ overload and increased miR-214-3p expression. In the animal model silencing Kcnq1ot1 and CASP-1 produced results consistent with in vitro outcome followed by improved cardiac function as ameliorated ejection fraction and fractional shortening [133].

HOTAIR is one of the first identified lncRNAs and it has a crucial role in the pathophysiology of CVDs [134]. Expression of HOTAIR was found significantly downregulated in myocardial tissues and serum of patients with DCM in comparison to patients with DM and healthy controls [135]. In the diabetic model of T1DM, the expression of HOTAIR was also found decreased, whereas its target miR-34a was found to be overexpressed [125]. On the other hand, the overexpression of HOTAIR protected against diabetes‐induced cardiac hypertrophy and dysfunction. Furthermore, diminished levels of fibrotic markers, such as TGF-β, col-I, col-III showed that HOTAIR treatment decreased cardiac fibrosis. Importantly, the inhibition of HOTAIR enhanced HG‐induced inflammation, oxidative stress, and apoptosis, whereas enhancing HOTAIR expression negated inflammation of STZ-induced DCM [125]. HOTAIR was found to function as a molecular sponge of miR‐34a which directly targeted SIRT1, a promising potential target for the treatment of CVD, and in particular DCM [125, 136].

**Fig. 2:**
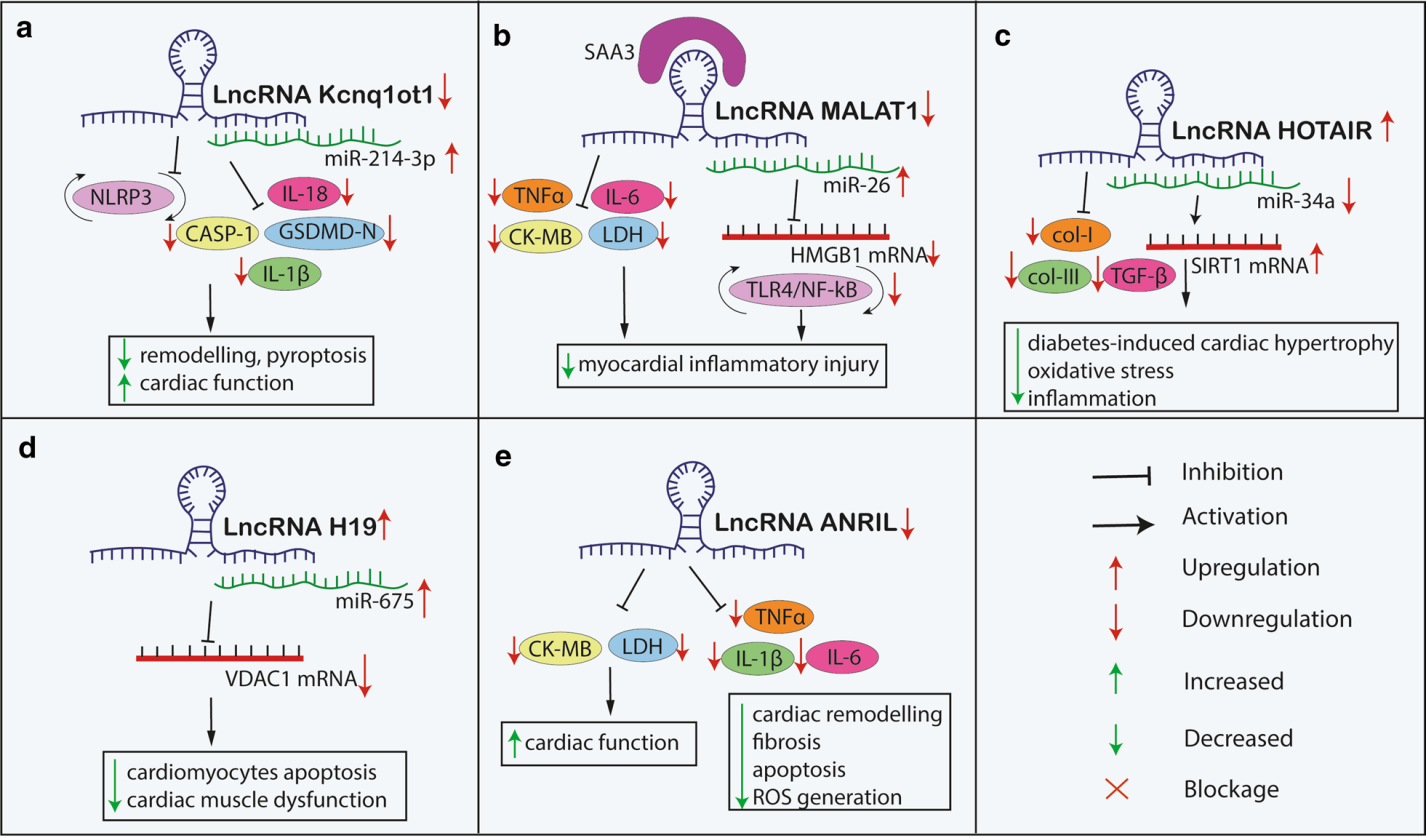
The possible therapeutic mechanism of lncRNAs-contributed to the oxidative stress, inflammation and cardiac function process in diabetic cardiomyopathy. *CASP-1* Caspase 1, *CK-MB* creatine kinase myocardial band, *GSDMD-N* N-terminal of gasdermin D, *HMGB1* high mobility group box 1, *HOTAIR* HOX transcript antisense intergenic RNA, *IL* interleukin, *Kcnq1ot1* KCNQ1 overlapping transcript 1, *LDH* lactate dehydrogenase, *LncRNA* long non-coding RNA, *MALAT1* metastasis-associated lung adenocarcinoma 1, *miR* microRNA, *mRNA* messenger RNA, *NLRP3* nod-like receptor protein 3, *NF-κB* nuclear factor kappa-light-chain-enhancer of activated B cells, *ROS* reactive oxygen species, SAA3 serum amyloid antigen 3, *SIRT1* Sirtuin 1, *TLR4* toll-like receptor 4, *TNFα* transforming growth factor β, *VDAC1* voltage-dependent anion channel 1

The above-mentioned studies indicate that the influence of LncRNAs on CASP-1, NLRP3, SIRT1 and TGF-β pathways presents as a new significant pathophysiology mechanism of cardiac remodeling in DCM and stands as a possible future therapeutic target (Fig. 2) (Table 2).

**Table 2:** Evaluating lncRNAs as a treatment approach in diabetic cardiomyopathy

Refs.	lncRNA	miRNA directly targeted by lncRNA	Regulated genes	Pathophysiological mechanism	Species, material, method	Conclusions
*Upregulated lncRNAs*						
[141, 142]	↑ANRIL	–	*CDH5* *HBEGF*	Inflammation Oxidative stress Apoptosis Fibrosis	STZ-induced diabetes model* In vitro, rats (Wistar rats, ANRILsiRNA group intraperitoneal injections of ANRIL siRNA or siRNA-negative control (NC) group *Diabetes was induced by intraperitoneal injection of STZ at 40 mg/kg per mouse. Mice with hyperglycemia (3 h FBG levels ≥ 16.7 mmol/l for 3 days were considered as having diabetes	ANRIL is upregulated in rat diabetic model. ANRIL siRNA treatment revealed improvement in cardiac function, LVEF, LVFS were elevated and LVEDD, LVPWD were decreased among diabetic rats. Diminishing ANRIL expression decreased myocardial inflammation as TNFα, IL‐6, IL‐1β levels were reduced, suppressed fibrosis via diminishing Col1, Col2 and alleviated apoptosis through downregulating caspase-3, Bax and upregulating Bcl-2, also diminished oxidative stress via decreasing ROS, and MDA.
[133]	↑Kcnq1ot1	miR-214-3p	*ADIPOR1 Kcnq1*	Inflammation Pyroptosis	T1DM STZ-induced diabetes model* In vivo*,* human blood serum, mice In vitro, human AC16 cells and mice cardiomyocytes (C57BL/6 mice and ventricular cardiomyocytes (AC16 cell line) and primary cardiomyocytes treated with 50 mmol/l glucose (HG) conditions were used. Cells were transfected with small interfering RNA (siRNA) against Kcnq1ot1 (si-Kcnq1ot1), miR-214-3p mimics (miR-214-3p), the anti-miRNA oligonucleotide of miR-214-3p (AMO-214-3p) or negative control (si-NC, NC, AMO-NC) using X-treme GENE siRNA Transfection Reagent *Diabetes was induced by an intraperitoneal injection of STZ 50 mg/kg/day for 5 days. Mice with glucose levels ≥ 16.7 mmol/l were considered as having diabetes	Kcnq1ot1 is overexpressed in serum of diabetic patients and in HG-induced cardiomyocytes. Silencing *Kcnq1ot1* inhibits cardiomyocytes pyroptosis by blocking CASP-1 via miR-214-3p
[132, 133]	↑Kcnq1ot1	miR-214-3p	*ADIPOR1 Kcnq1*	Inflammation Pyroptosis Fibrosis	STZ-induced diabetes model* In vivo, human blood serum In vivo*, *in vitro*,* mice (C57BL/6 mice, cardiomyocytes incubated with 30 mmol/l glucose (HG) for 24 h were used), Cells were transfected with small interfering RNA (siRNA) against Kcnq1ot1 (si-Kcnq1ot1), miR-214-3p mimics (miR-214-3p), the anti-miRNA oligonucleotide of miR-214-3p (AMO-214-3p) or negative control (si-NC, NC, AMO-NC) using X-treme GENE siRNA Transfection Reagent *Diabetes was induced by an intraperitoneal injection of STZ 50 mg/kg/day for 5 days. After 72 h, mice with blood glucose of > 16.7 mmol/l were considered as having diabetes	*Kcnq1ot1* is increased in LV tissue of diabetic mice and corresponds with myocardial dysfunction progression. Knockdown of *Kcnq1ot1* by a lentivirus-shRNA resulted in enhanced cardiac function. *Kcnq1ot1* silencing stimulated gasdermin D cleavage and IL-1β expression and therefore suppressed TGF-β1/smads pathway via miR-214-3p and CASP-1 in HG-induced CFs
[124]	↑MALAT1	miR-26a	*HMGB1*	Apoptosis	In vitro*,* human adult ventricular cardiomyocytes (AC16 cell line)* *AC16 cells were treated with 300 ĶM palmitic acid to induce myocardial lipotoxic injury transfected with siRNA (negati, miR-26a mimics, NC mimics, miR-26a inhibitor, NC inhibitor, and TNF-α-specific siRNA (si-TNF-α)	MALAT1 overexpression is promoted by SFAs. MALAT1 directly binds to miR-26a. Silencing of MALAT1 diminished cardiac inflammatory injury via modulating miR-26a/*HMGB1*/TLR4/NF-κB signaling pathway, suggesting MALAT1 capability to regulate inflammatory processes
[140]	↑MALAT1	–	*SAA3*	Inflammation	STZ-induced diabetes model* In vivo*, *in vitro*,* mice kidney tissues *(*HUVECs incubated in 25 mM D-Glucose (HG) condition) *Diabetes was induced by an intraperitoneal injection of STZ (70 mg/kg of mice) × 3 on alternate days	Hyperglycemia stimulates MALAT1 expression and *SAA3* in HUVECs. Silencing of MALAT1 and *SAA3* expression alleviated the inflammation process via the downregulation of IL-6 and TNFa levels. MALAT1 is upregulated in the kidneys of diabetic mice. MALAT1 directly targets the *SAA3* gene, which is a key regulator of inflammatory cascade
[128]	↑NONRATT007560.2	miR-7a-1-3 miR-92a-2-5p miR-208a-5p miR-208b-5p miR-3065-5p miR-3575	–	Oxidative stress Apoptosis	HG- induced diabetes model* In vitro, Sprague .Dawley rats, cardiomyocytes incubated with 5 mmol/l D-glucose (NG) or 33 mmol/l D-Glucose (HG)	HG stimulated cardiomyocytes demonstrated elevated levels of NONRATT007560.2 and induced apoptosis Silencing of NONRATT007560.2 suppressed apoptosis and oxidative stress via decreasing ROS in rat cardiomyocytes
t						
[127]	↓H19	miR-675	*VDAC1*	Inflammation Apoptosis	STZ-induced diabetes model* In vitro, primary neonatal rats cardiomyocytes transfected with miR-30d mimic, and negative control (NC) using X-treme Gene siRNA Transfection Reagent in silico*,* in vivo*,* Wistar rats, *Diabetic rats were injected intraperitoneally with 35 mg/kg/d of STZ for 3 days. Fasting blood glucose levels were measured 72 h after STZ injection and diabetes was considered successfully established in rats with blood glucose levels > 16.7 mmol/l	HG exposure reduced H19 expression. Overexpression of H19 improved LV function in diabetic rats by diminishing oxidative stress, inflammation and apoptosis. H19 impacts myocardial apoptosis in DCM. H19 decreased myocardial apoptosis via miR-675 which directly targeted apoptosis-related *VDAC1* gene
[125]	↓HOTAIR	miR-34a	*SIRT*	Inflammation Oxidative stress Apoptosis Fibrosis	T1DM STZ-induced diabetes model* In vivo*,*mice In vitro*,* mice, rats cardiomyocytes (Male C57/B6 mice were given a single injection of adeno-associated virus (AAV) 2-HOTAIR) H9c2 cells were exposed to high glucose and infected with shHOTAIR for 4 h Mice cardiomyocytes were exposed to a HG concentration (33 mM glucose) and transfected with miR-34a mimics, antagomiR-34a, and their control were synthesized *Diabetes was induced by intraperitoneal injection of STZ at 50 mg/kg per mouse for 5 consecutive days. Mice with hyperglycemia (3 h FBG levels ≥ 250 mg/dl) were considered as having diabetes	Decreased HOTAIR stimulated HG-induced oxidative stress, inflammation, apoptosis in STZ-treated mice. HOTAIR overexpression downregulates levels of fibrotic markers

↑ / ↓indicates the up/down regulation of the lncRNAs determined in the HG conditions / diabetic heart model

*ADIPOR1* adiponectin receptor 1, *ANRIL* antisense noncoding RNA gene at the INK4 locus, *Bcl2* B-cell lymphoma 2, *Bax* Bcl-2 Associated X-protein, *CDH5* Cadherin 5, *CFs* cardiac fibroblasts, *CASP-1* caspase-1, *Col1* collagen type I, *Col2* collagen type II, *DCM* diabetic cardiomyopathy, *dl* decilitre, *GFP* green fluorescent protein, *H19* imprinted maternally expressed transcript, *HBEGF* Heparin Binding EGF Like Growth Factor, *HF* heart failure, *HG* high-glucose, *HMGB1* high mobility group box 1 protein, *HOTAIR* HOX Transcript Antisense Intergenic RNA, *HUVECs* human umbilical vein endothelial cells, *IL-1β* interleukin-1β, *IL-6* Interleukin-6, *Kcnq1* potassium voltage-gated channel subfamily Q Member 1, *LV* left ventricle, *LVEDD* left ventricular end diastolic dimension, *LVEF* left ventricular ejection fraction, *LVFS* left ventricular fractional shortening, *LVPWD* left ventricular posterior wall thickness at end-diastole, *Kcnq1ot1* KCNQ1 overlapping transcript 1, *kg* kilogram, *L* litre, *MALAT1* Metastasis Associated Lung Adenocarcinoma Transcript 1, *mg* milligram, *MDA* malondialdehyde, *mM* millimolar, *mmol* millimole, *miR* microRNA, *mTOR* mammalian target of rapamycin, *NC* negative control, *NG* normal glucose, *NF-κB* nuclear factor kappa-light-chain-enhancer of activated B cells, *PA* palmitic acid, *ROS* reactive oxygen species, *SAA3* serum amyloid A3, *SFAs* saturated fatty acids, streptozocin, *shRNA* short hairpin RNA, *Sirt* sirtuin, *siRNA* small interfering RNA, *STZ* streptozocin, *T1DM* type 1 diabetes mellitus, *TGF‐β* transforming growth factor β, *TLR4* toll-like receptor 4, *TNFα* tumor necrosis factor alpha, *TRAF* TNF receptor associated factor, *VDAC1* voltage-dependent anion channel 1

### NF-κB and TNF signaling pathways are involved in cardiomyocyte injury mediated by LncRNAs

Myocardial damage resulting from an imbalance in the ROS generation and inflammation might be a consequence of DM associated hyperglycemia or hyperlipidemia [137]. For example, the excess saturated fatty acids, especially palmitic acid (PA), found in patients with DM may deposit in cardiomyocytes and cause lipotoxic injury [138]. Cardiomyocytes treated with PA were found to produce significantly upregulated inflammatory factors TNFα and IL-1β, along with MALAT1, which belongs to the lncRNA family. Recently MALAT1 has been reported to play a crucial role in cardiomyocytes ischemia reperfusion damage [139]. On the other hand, the silencing of MALAT1 expression decreased the range of myocardial injury by reducing biomarkers of myocardial damage, lactate dehydrogenase (LDH) and CK-MB, as well as by increasing sponge-miR-26 expression [124]. What is important, miR-26 inhibits TLR4/NF-κB inflammatory signaling pathway by binding to its target gene, *HMGB1*. Furthermore, manipulation of MALAT1/miR-26 expression revealed the potential role of these molecules in ameliorating PA-induced cell death via TNFα apoptosis pathway. MALAT1 inhibition results in the alleviation of SFA-induced myocardial inflammatory injury via the miR-26a/HMGB1/TLR4/NF-κB axis [124].

In HG conditions ECs exhibit markedly upregulated both MALAT1 expression and SAA3, inflammatory ligand, and target of MALAT1 [140]. Those changes are followed by the increased expression of inflammatory markers, i.e. IL-6 and TNFα. The silencing of MALAT1 was found to result in the reduction of IL-6 and TNFα expression, even in the presence of SAA3, but cytokine levels were reversed only partially. It indicates that there are other pathways than MALAT1-SAA3 that play a role in HG stress regulation [140].

ANRIL is located at locus with the strongest genetic susceptibility for CVD in the chromosome 9p21 region and was shown to control the expression of *CDH5* and *HBEGF* gene involved in vascular permeability, leukocyte transmigration and inflammation [141]. ANRIL was found to be upregulated along with elevated levels of TNFα in the myocardium of diabetic rats, which suggests its association with the development of DCM [142]**.** ANRIL silencing leads to the decreased levels of CK-MB and LDH, which indicates that interference of ANRIL expression can improve cardiac function index. Moreover, plasma levels of TNFα, IL-6 and IL-1β levels were found to be significantly elevated in the diabetic model, though ANRIL expression interference diminished the level of these cytokines. Novel ANRIL-based therapeutic strategies can offer a promising approach to inhibit cardiomyocytes fibrosis, apoptosis and ROS generation in DCM treatment [142]**.**

By and large, the downregulation of MALAT-1 decreases the range of myocardial lipotoxic injury and reduces inflammation under HG conditions. Interfering ANRIL expression with siRNA alleviates myocardial remodeling in diabetic rats, and decreases the level of inflammatory cytokines including TNFα. Subsequent studies should answer whether MALAT1 and ANRIL can serve as a drug target in chronic diabetic complications (Fig. 2) (Table 2).

### LncRNAs targeting HMGA1

High-mobility group AT-hook 1 (HMGA1) is a key partaker in cardiovascular complications of diabetes, both at the vascular and the cardiac level [143, 144]. HMGA1 expression is modulated by miRNAs known to be involved in cardiovascular disease, such as Let-7 and miR-26a [145]. More recently, a number of lncRNAs are emerging as regulators of HMGA1. *SNHG1,* the expression of which is induced by oxygen deprivation, mediates cardiomyocyte hypertrophy via targeting miR-15a/HMGA1 [146–148]. *HOTTIP* has been shown to regulate the miR-218/HMGA1 axis [149, 150]. This is particularly interesting, as both HMGA1 and miR-218 are involved in the development of cardiovascular complications in diabetes [24, 151]. LncRNA GACAT3 acts as a competing endogenous RNA of HMGA1 [152]. The increased understanding of the modulation of HMGA1 expression might pave the way to both diagnostic and therapeutic applications (Fig. 2) (Table 2).

## Current perspectives and limitations

**Fig. 3:**
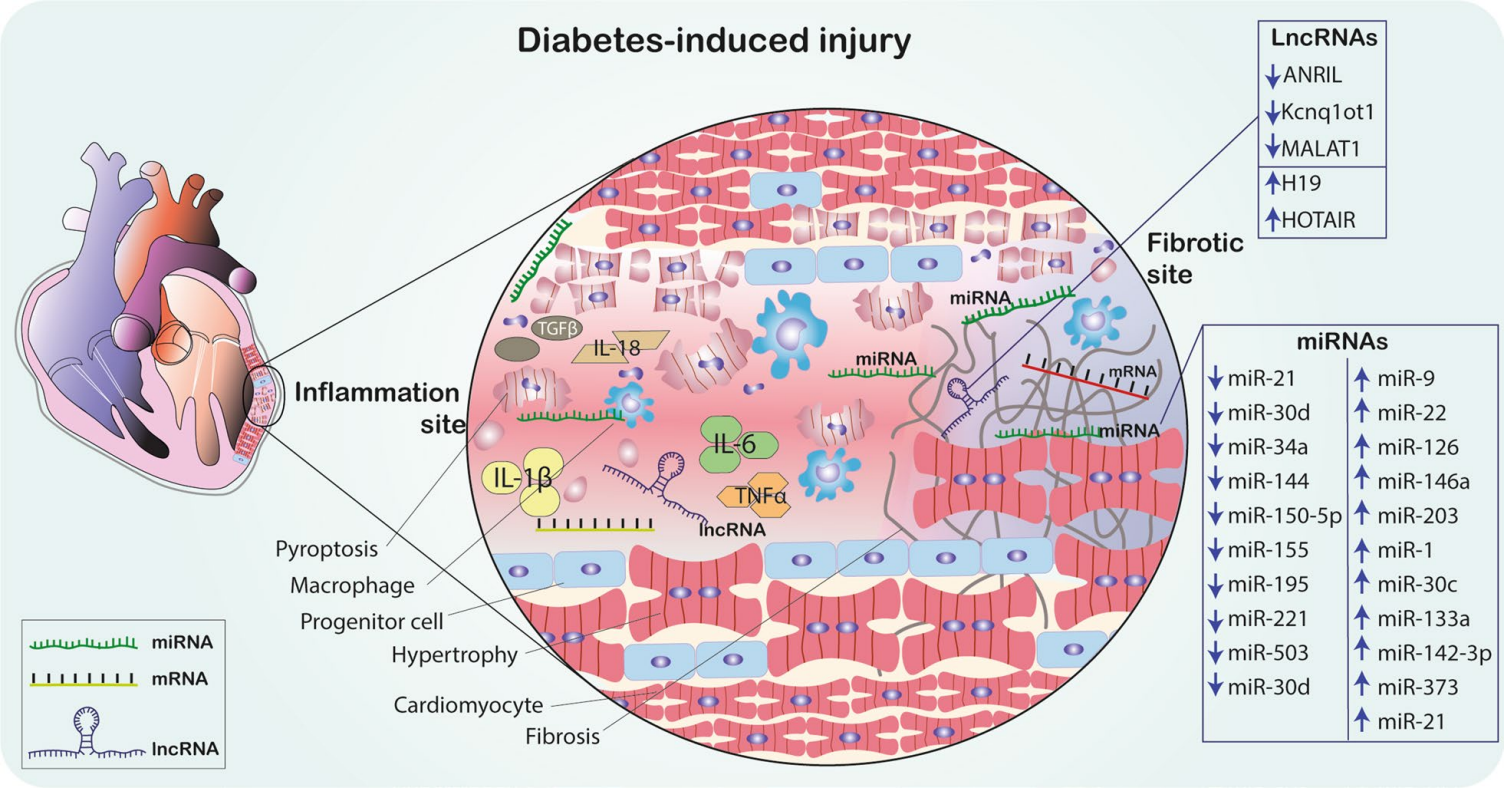
MiRNAs and lncRNAs as therapeutics in diabetes-induced cardiomyopathy. ↑ indicates the mimic-use as treatment, ↓ indicates inhibitor-use as treatment against diabetic cardiomyopathy. A*NRIL* antisense non-coding RNA in the INK4 locus, *H19* imprinted maternally expressed transcript, *HOTAIR* HOX transcript antisense intergenic RNA, *IL* interleukin, *Kcnq1ot1* KCNQ1 overlapping transcript 1, *lncRNA* long non-coding RNA, *MALAT1* metastasis-associated lung adenocarcinoma 1, *miRNA* microRNA, miR; *mRNA* messenger RNA, *TGFβ* transforming growth factor β, *TNFα* tumor necrosis factor-alpha

Numerous studies have shown that miRNAs and lncRNAs could act as potential biomarkers as well as novel treatments in DCM. In Fig. 3 miRNAs/lncRNAs as potential therapeutics was presented.

**Fig. 4:**
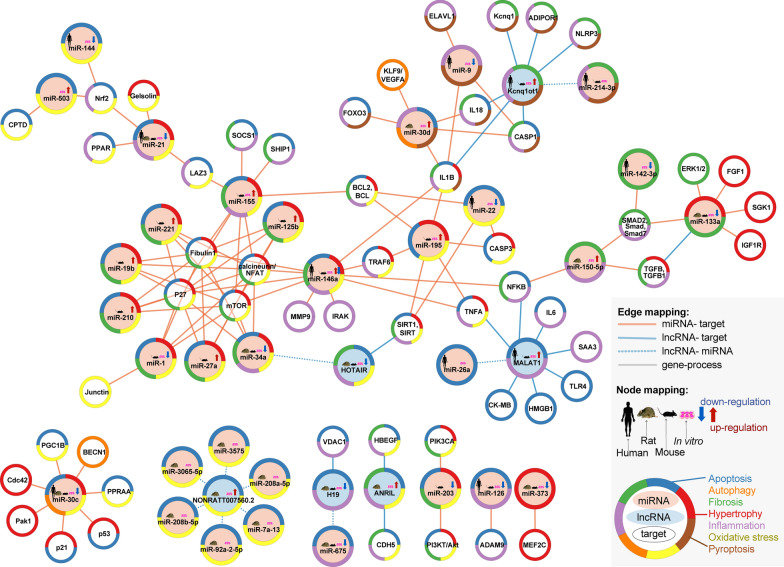
Summarizing graph showing information from the studies evaluating miRNAs and lncRNAs as potential biomarkers in diabetic cardiomyopathy. The figure was generated using data from the published literature regarding ncRNAs, their targets and affected biological processes. Literature data (Figs. 1, 2, Tables 1 and 2) were transformed into a tabular network file. The miRNA, lncRNA and their targets are shown as nodes and connections between them as edges. Visualization of the network was performed using Cytoscape v3.7.2. and the Perfuse force layout algorithm. Additional information regarding affected biological processes retrieved from publications, model organisms and ncRNAs expression changes shown in the studies was used for visual mapping for the nodes

To summarize and present the published data of miRNAs and lncRNAs involved in pathophysiology of DCM, we have generated Fig. 4 regarding ncRNAs and their target genes, affecting biological processes. Literature data (Tables 1 and 2) was transformed into a tabular network file. Cardiac fibrosis, hypertrophy, oxidative stress, inflammation, apoptosis, autophagy, and pyroptosis were presented. The miRNA, lncRNA and their targets are shown as nodes and connections between them as edges. Visualization of the network was performed using Cytoscape v3.7.2. Additional information regarding model organisms and ncRNAs expression changes shown in the studies was used for visual mapping of the nodes. According to this network (Fig. 4), we can conclude that miR-146a and miR-195 appear to be the most promising miRNAs as regulators in DCM, since those miRNAs placed in the center of the network, can target at least six different genes and can regulate different biological processes involved in DCM, such as inflammation, hypertrophy, apoptosis, or oxidative stress (confirmed in the literature by experimental analysis). Furthermore, to date most of the studies have aimed to analyze the impact of lncRNAs MALAT1 and Kcnq1ot1 in DCM. MALAT1 was found to be associated with inflammation and apoptosis processes, whereas Kcnq1ot1 association was identified with pyroptosis, cardiac fibrosis, and inflammation. Importantly, as it is presented in Fig. 4 those lncRNAs can regulate at least 6 different genes and can sponge miRNAs. Important to note that miR-146a and those lncRNAs (MALAT1 and Kcnq1ot1) were studied not only in the in vitro and in vivo analysis, but were also demonstrated in human studies [71, 124, 132, 133].

On the other hand, as it is summarized in Fig. 4, several studies have shown that the upregulation of ANRIL and downregulation of HOTAIR may have consequences in the form of cardiac fibrosis, oxidative stress, apoptosis and inflammation processes in DCM. Therefore, future studies should focus more on these lncRNAs (HOTAIR and ANRIL) as their therapeutic potential may be higher than that demonstrated in the current literature.

Measuring their expression in blood components and heart tissue may improve the diagnosis and prediction of adverse cardiac outcomes. However, the use of lncRNAs and miRNAs as biomarkers in clinical practice still faces many limitations: (i) a small number of human studies describing the role of ncRNAs in the processes of oxidative stress and inflammation in DCM; (ii) many of the studies described in this review require further validation and assessment of their results reproducibility; (iii) a number of studies that analyzed the importance of ncRNAs in DCM used STZ-induced diabetes, which resembles T1DM more closely than T2DM. Due to the different pathophysiology of T1 and T2 diabetes, further research is needed to clarify the differences in expression pattern of individual miRNAs/lncRNAs related to processes underlying DCM in T1DM and T2DM. (iv) individual molecules examined in diabetic patients such as miR-223 and miR-126 are not specific to this disease only; (v) although similar expression of single lncRNAs and miRNAs in DCM has been confirmed independently by various authors, describing and validation of specific biomarker signature patterns in DCM remain challenging**.**

Compared to other types of drugs, ncRNA therapies excel in several aspects. Due to the development of bioinformatics tools including in silico prediction analysis and the simple structure of ncRNAs, the mechanism of action of these molecules can be predictable. The use of miRNAs in therapy allows for the simultaneous targeting of several protein-coding genes. Moreover, upregulation or downregulation of miRNAs expression to their physiological concentrations allows the restoration of homeostasis [153]. Importantly, ncRNAs are able to target genes that are still “undruggable” and unlike regular medications used today, they have not been found to be affected by drug resistance [154].

The use of ncRNA as a therapeutic agent is a promising approach with the possibility of treating a wide range of human diseases at the molecular level [155]. It is worth mentioning that the delivery systems that are successful in the in vivo studies differ structurally and chemically. In each particular case, special forms of delivery of ncRNAs may be necessary to achieve the best efficacy without causing harmful side effects. Lipid nanoparticles (LNP) seem to be a promising and effective way for ncRNA therapy [153]. For example, inclisiran in LNP formulation as a small interfering RNAs (siRNA) against PCSK9 is used for a gene therapy of primary hyperlipidemias [156, 157]. Alternatively, neutral or synthetic polymers may be applied for ncRNA therapy. It was shown on an animal model that in post-infarcted heart intracoronary injection of an antagomiR-92 encapsulated in poly(lactic-co-glycolic acid) stimulated angiogenesis, improved myocardial function and prevented against adverse infarct remodeling [158]. Moreover, the use of exosomes, which are extracellular vesicles released by cells, appears to be promising as well. It has been shown that due to their favorable pharmacokinetic properties, exosomes can serve as an attractive carrier of nucleic acids capable of penetrating physiological barriers inaccessible to other drug carriers [159, 160]. However, much work is still needed in this field. Nevertheless, the potential clinical impact is undoubtedly worth the investment.

## Conclusion

Several studies highlighted the promising role of these molecules as potential therapeutic targets in miRNA- and lncRNA-based novel treatments. As described above, this therapeutic approach may consist in the inhibition by means of antagonists or restoration of loss of function molecules with mimics that are similar to endogenous molecules. Yet, the detailed mechanism of action of the described miRNAs and lncRNAs on cardiomyocytes oxidative stress and inflammation has not been fully explained and more studies need to be conducted. Importantly, a single miRNA or lncRNA may target multiple genes, thus understanding the miRNA–lncRNA interaction network and functions, as well as creating an effective and inexpensive way of delivering therapeutics are prerequisites to apply therapy in the future. A subsequent comprehensive in silico analysis may provide novel information of signaling pathways involved in pathological changes in DCM and identify miRNAs and lncRNAs that could potentially serve as therapeutic targets [25, 27, 161].

## Data Availability

Not applicable.

## Abbreviations

α2M: Alpha-2-macroglobulin
α‐SMA: Alpha-smooth muscle actin
AA: Amyloid A
ADAM9: ADAM metallopeptidase domain 9
ADAMs: Human A disintegrin and metalloproteases
Adipor1: Adiponectin receptor 1
AMI: Acute myocardial infarction
ANRIL: Antisense non-coding RNA in the INK4 locus
AP: Amyloid P
AP-1: Activator protein 1
APPs: Acute-phase proteins
ARC: Activity regulated cytoskeleton associated protein
Bcl-2: B-cell lymphoma 2
CAD: Coronary artery disease
CASP: Caspase
CDH5: Cadherin 5
CK-MB: Creatine kinase myocardial band
Col1α1: Collagen type I alpha 1
Col4α1: Collagen type IV alpha 1
Col‐I: Collagen type-I
Col‐III: Collagen type-III
CPDT: 5,6 Dihydrocyclopenta-1,2-dithiole-3-thione
CRP: C-reactive protein
CTGF: Connective tissue growth factor
CVDs: Cardiovascular diseases
DCM: Diabetic cardiomyopathy
DM: Diabetes mellitus
DNA: Deoxyribonucleic acid
ECM: Extracellular matrix
ECs: Endothelial cells
ELAVL1: ELAV like protein 1
EMT: Endothelial-to-mesenchymal transition
ERK1/2: Extracellular signal-regulated kinases 1 and 2
FGF1: Fibroblast growth factor 1
FN: Fibronectin
FOXO3a: Forkhead box O3
GACAT3: Gastric cancer-associated transcript 3
GSDMD-N: N-terminal of gasdermin D
H19: Imprinted maternally expressed transcript
HAECs: Human aortic endothelial cells
HBEGF: Heparin binding EGF like growth factor
HF: Heart failure
HG: High glucose
HMGA1: High-mobility group AT-hook 1
HMGB1: High mobility group box 1
HO-1: Heme oxygenase 1
HOTAIR: HOX transcript antisense intergenic RNA
HOTTIP: HOXA transcript at the distal tip
I/R: Ischemia/reperfusion
IL: Interleukin
IRAK1: Interleukin-1 receptor-associated kinase 1
Kcnq1: Potassium voltage-gated channel subfamily Q member 1
Kcnq1ot1: Potassium voltage-gated channel subfamily Q member 1 overlapping transcript 1
LAZ3: Lymphoma-associated zinc finger 3
LDH: Lactate dehydrogenase,
lncRNAs: Long non-coding RNAs
LNP: Lipid nanoparticle
MALAT1: Metastasis-associated lung adenocarcinoma 1
MAPK: Mitogen-activated protein kinase
MCP-1: Monocyte chemoattractant protein-1
MDA: Malondialdehyde
Mef2c: Myocyte enhancer factor 2C
MMP-9: Matrix metalloproteinase 9
mRNA: Messenger RNA
miRs: MicroRNAs, miRNAs
ncRNA: Non-coding RNA
NF-κB: Nuclear factor kappa-light-chain-enhancer of activated B cells
NLRP3: Nod-like receptor protein 3
NLRP3: NLR family pyrin domain containing 3
Nrf2: Nuclear factor erythroid 2-related factor 2
Nt: Nucleotides
PA: Palmitic acid
PCSK9: Proprotein convertase subtilisin/kexin type 9
PGC-1α: Peroxisome proliferator-activated receptor gamma coactivator 1-alpha
PGC-1β: Peroxisome proliferator-activated receptor gamma coactivator 1-bet
phospho-Akt: Phosphorylated protein kinase B
phospho-eNOS: Phosphorylated endothelial nitric oxide synthase
PI3K/Akt: Phosphoinositide 3-kinase and protein kinase B
PIK3CA: Phosphoinositide 3-kinase catalytic subunit alpha
PPARα: Peroxisome proliferator-activated receptor-alpha
p-Smad2: Phosphorylated SMAD family member 2
p-Smad3: Phosphorylated SMAD family member 3
PDCD4: Programmed cell death 4
RNA: Ribonucleic acid
ROS: Reactive oxygen species
SAA3: Serum amyloid antigen 3
siRNA: Small interfering RNA
Sirt1: Sirtuin 1
Smad7: SMAD family member 7
SNHG1: Small nucleolar RNA host gene 1
SOD: Superoxide dismutase
STZ: Streptozocin
T1DM: Type 1 diabetes mellitus
T2DM: Type 2 diabetes mellitus
TGF-β1: Transforming growth factor β1
TGF-β3: Transforming growth factor β3
TLR4: Toll-like receptor 4
TNFα: Tumor necrosis factor-alpha
TRAF6: TNF Receptor associated factor 6
VDAC1: Voltage-dependent anion channel 1
